# Functional identification of a *Streptomyces lividans* FKBP-like protein involved in the folding of overproduced secreted proteins

**DOI:** 10.1098/rsob.190201

**Published:** 2019-10-30

**Authors:** R. L. Vicente, S. Marín, J. R. Valverde, C. Palomino, R. P. Mellado, S. Gullón

**Affiliations:** 1 Departamento de Biotecnología Microbiana, Centro Nacional de Biotecnología (CNB-CSIC), c/Darwin 3, 28049 Madrid, Spain; 2 Scientific Computing Service, Centro Nacional de Biotecnología (CNB-CSIC), c/Darwin 3, 28049 Madrid, Spain

**Keywords:** *Streptomyces lividans*, secretory protein folding, peptidyl-prolyl *cis/trans* isomerases, FKBP

## Abstract

Some bacterial peptidyl-prolyl *cis/trans* isomerases (PPIases) are involved in secretory protein folding after the translocation step. *Streptomyces lividans* has been used as a host for engineering extracellular overproduction of homologous and heterologous proteins in industrial applications. Although the mechanisms governing the major secretory pathway (Sec route) and the minor secretory pathway (Tat route) are reasonably well described, the function of proteins responsible for the extracellular secretory protein folding is not characterized as yet. We have characterized a Tat-dependent S*. lividans* FK506-binding protein-like lipoprotein (FKBP) that has PPIase activity. A mutant in the *sli-fkbp* gene induces a secretion stress response and affects secretion and activity of the Sec-dependent protein α-amylase. Additionally, propagation in high copy number of the *sli-fkbp* gene has a positive effect on the activity of both the overproduced α-amylase and the overproduced Tat-dependent agarase, both containing proline *cis* isomers. Targeted proteomic analyses showed that a relevant group of secreted proteins in *S. lividans* TK21 are affected by Sli-FKBP, revealing a wide substrate range. The results obtained indicate that, regardless of the secretory route used by proteins in *S. lividans*, adjusting the expression of *sli-fkbp* may facilitate folding of dependent proteins when engineering *Streptomyces* strains for the overproduction of homologous or heterologous secretory proteins.

## 1. Background

Streptomycetes are Gram-positive soil bacteria that secrete an array of hydrolytic enzymes [1,2], among other metabolites, to ensure their survival in this harsh environment. This natural capacity to produce hydrolytic enzymes in industrial applications has attracted great interest in using streptomycetes as hosts for the production of such extracellular proteins.

*Streptomyces lividans* is a bacterium with a relaxed restriction modification system, which facilitates its transformation by heterologous DNA. The genome sequence of *S. lividans* is known [3,4], and the bacterium has been largely used as a host for the production of engineered secretory proteins of high commercial value [5,6].

Extracellular protein secretion across the *S. lividans* cytoplasmic membrane mainly uses the major secretion pathway (Sec) and the minor twin arginine translocation (Tat) secretion pathway [7]. The Sec pathway targets newly synthesized pre-proteins to the membrane using the signal recognition particle (SRP), which interacts with the pre-protein signal peptides, and its membrane receptor FtsY [8]. The ribosome–protein complex may also interact with the translocase complex SecYEG for protein secretion. Sec-secreted proteins are released into the medium in an unfolded conformation. In contrast, proteins secreted by the Tat pathway appear to be exported outside the cell fully folded [9]. Thus, the Tat route is an appealing secretion system in streptomycetes where up to 27 proteins have been confirmed to be secreted by the *Streptomyces coelicolor* Tat system [10]. In *S. lividans*, the Tat system has three components (TatA, TatB and TatC), and the Tat signal peptide, containing the highly conserved twin-arginine motif SRRXFLK, at its amino end, is thought to be recognized by the TatA–TatB heterocomplex [11]. This complex theoretically interacts with TatC in the membrane, and, then, oligomerization of TatA forms the membrane pore to support secretion of the Tat proteins [11]. In *Escherichia coli* the signal peptide recognition is mediated by the TatB–TatC complex [12].

The main enzymes involved in extracellular folding of secretory proteins are the thiol-disulfide oxidoreductases and the peptidyl-prolyl *cis/trans* isomerases (PPIases). Bacterial PPIases have been linked to protein folding and secretion because of their capacity to catalyse the *cis/trans* isomerization of peptide bonds preceding prolyl residues [13]. The PPIases typically belong to three functional groups, cyclophilins, the FK506-binding proteins (FKBPs) and parvulins, and are ubiquitously distributed among bacteria [13].

*Bacillus subtilis* PrsA is a lipoprotein that belongs to the parvulin family and is considered an important factor for protein secretion. The cellular level of PrsA and the rate of secretion have been shown to be interrelated in *B. subtilis.* Depletion of PrsA leads to a reduction of the levels of secreted heterologous α-amylase (AmyQ) [14] as well as of the amount of several endogenous secretory proteins [15]. PrsA overproduction strongly increases the production of AmyQ or the SubC protease [16].

No equivalent PPIases have been experimentally characterized to exert this role in *S. lividans* yet. In the present work, we characterize a Tat-dependent *S. lividans* FKBP-like lipoprotein (Sli-FKBP) that positively affects the production of a significant number of Sec- and Tat-secreted proteins containing putative proline *cis* isomers in *S. lividans* TK21.

## 2. Methods

### 2.1. Bacterial strains, plasmids and media

The *S. lividans* TK21 wild-type strain [17] and its derivatives were cultured in liquid NMMP medium in the presence of mannitol as a carbon source [18]. Apramycin (50 µg ml^−1^), thiostrepton (50 µg ml^−1^), kanamycin (50 µg ml^−1^) and chloramphenicol (25 µg ml^−1^) were added to the R5 and MS solid media, when required.

### 2.2. Construction of gene disruption mutant

To construct the *S. lividans* Δ*sli*-*fkbp* mutant strain oligonucleotides FKBPdisFw (5′-GGGCTGCAGAACAGCTACGACCGCAAGAC-3′) and FKBPdisRv (5′-GCCTCTAGACACACCCTTGTACTGCACGA-3′) were used to amplify a 420 nt long DNA fragment that was inserted into the non-replicating suicide plasmid pOJ260 of *Streptomyces* [19] through its unique *Xba*I and *Pst*I sites to generate plasmid pOJFKBP. The plasmid was used to conjugate *E. coli* to *Streptomyces* as described [20], to inactivate the chromosomal copy of *sli-fkbp* by insertion of pOJ260 by single homologous recombination between pOJFKBP and the *sli-fkbp* copy in the chromosome. *Escherichia coli* ET12567 carrying the non-transmissible ‘driver’ plasmid pUZ8002 was used for conjugation [21]. Apramycin-resistant strains containing the disrupted gene *sli-fkbp* were selected upon verification of the disruption by polymerase chain reaction (PCR) amplification (not shown). Plasmids pAMI11 [22] and pAGAs5 [23] carrying the *S. lividans* gene *amlB* or the *S. coelicolor* agarase gene *dagA*, respectively, were used to transform the *S. lividans* TK21 and *S. lividans* Δ*sli-fkbp*.

### 2.3. Overexpression of FKBP

The oligonucleotides FKBP-HindIII (5′-TTTTAAGCTTTCTCACGCCGTAGAGTTGC-3′) and FKBP-XbaI (5′-TTTTTCTAGACCCTCCCAGATGTCCTTGAT-3′) containing the *Hin*dIII and *Xba*I sites and FKBP-P (5′-TTTGGATCCTTCTCACGCCGTAGAGTTGC-3′) and FKBP-T (5′-TTTTTGGATCCCCCTCCCAGATGTCCTTGAT-3′) containing the *Bam*HI sites were used to amplify the gene *sli-fkbp* and its possible promoter region. Chromosomal DNA of *S. lividans* TK21 strain was used as a template. The obtained 1222 bp long DNA fragments were subsequently cloned into the pGEM-T Easy vector (Promega) and sequenced. The plasmid pGEM, harbouring *sli-fkbp*, was digested with *Not*I to retrieve the 1244 bp long DNA fragment containing *sli-fkbp*; this was inserted into the multi-copy cosmid pFDT [24] through its unique *Not*I site to generate pFDTFKBP. Additionally, the pGEM harbouring *sli-fkbp* was digested with *Bam*HI, and the 1222 bp long DNA fragment was cloned into the multicopy plasmid pIJ487 previously digested with *Bam*HI to generate plasmid pIJFKBP.

Protoplasts from *S. lividans* TK21, *S. lividans* TK21(pAMI11) and *S. lividans* TK21(pAGAs5) were transformed with cosmid pFDTFKBP to obtain the *S. lividans* (pFDTFKBP), *S. lividans* TK21(pAMI11) (pFDTFKBP) and *S. lividans* TK21(pAGAs5) (pFDTFKBP) strains. Cosmid pFDT was propagated in *S. lividans* TK21, *S. lividans* TK21(pAMI11) or *S. lividans* TK21(pAGAs5) to generate the corresponding isogenic strains.

Protoplasts from *S. lividans* TK21 were transformed with plasmid pIJFKBP to obtain *S. lividans* (pIJFKBP). Plasmid pIJ487 was propagated in *S. lividans* TK21 to generate the corresponding isogenic strain.

### 2.4. Quantitative real-time polymerase chain reaction

Total RNA was isolated from bacteria growing cultures at different phases of growth (24 h, 36 h, 48 h) using the RNeasy midi Kit (Qiagen). Cell lysates were extracted twice with phenol–chloroform before being loaded onto RNeasy midi-columns for RNA purification. DNA, potentially contaminating the RNA preparations, was removed by incubation with RNase-free DNAse (Ambion) and its absence was tested by quantitative real-time PCR (qRT-PCR) amplification in the absence of reverse transcriptase. Complementary DNA was synthesized using the High Capacity Archive kit (Applied Biosystems). qRT-PCR was performed using SYBR Green technology in an ABI Prism 7300 Sequence Detection System (Applied Biosystems). Samples were initially denatured by heating at 95°C for 10 min. A 40-cycle amplification and quantification programme was then followed (95°C for 15 s and 60°C for 1 min) by a single fluorescence measurement per cycle, according to the manufacturer's recommendations. Subsequently, a final extension cycle (72°C, 1 min) was performed. Three biological samples from the different bacterial cultures were amplified in triplicate in separate PCR reactions. All PCR products were between 50 and 150 bp in length.

A melting curve analysis was conducted after amplification to distinguish the targeted PCR products from the non-targeted ones. The melting curves were obtained by heating at temperatures ranging from 60°C to 95°C at a rate of 0.2°C per second, with continuous fluorescence scanning. The *hrdB* transcript was carried out as an internal control to quantify the relative expression of the target genes. The *hrdB* transcript was used as a reference to normalize the relative expression of *Streptomyces* genes. The oligonucleotides used as primers to amplify the transcripts of two-component system *cssRS* and *tatC* genes were described previously [25,26]. Oligonucleotides SLI1639 FW (5′-GGACGAAGTTCGACGAGAAG-3′) and SLI1639 RV (5′-AGACCTGGCCGAGGTAGTT-3′) were used to amplify the *sli-fkbp* transcript.

### 2.5. Protein analysis and western blot experiments

Supernatants from the *sli-fkbp* mutant and the Sli-FKBP overproducer strains containing the multi-copy plasmids pAMI11 and pAGAs5 grown in NMMP medium [27] were processed as described [25]. For western blot analysis, extracellular proteins were fractionated by sodium dodecylsulfate–polyacrylamide gel electrophoresis (SDS-PAGE) in 10% and 12% (w/v) acrylamide gel [28]. An equivalent amount of protein loaded onto the SDS-PAGE acrylamide gel was corrected by the bacterial dry weight in each case.

Gel-fractionated proteins were transferred onto Immobilon polyvinylidene difluoride membranes (Millipore), as described [29]. The transferred material was incubated with polyclonal antibodies raised against *S. lividans* TK21 AmlB (a gift from C. Isiegas) and *S. coelicolor* agarase (DagA; [30]) followed by incubation with horseradish peroxidase-conjugated protein A (Invitrogen Laboratories) as described before [25].

### 2.6. Enzyme activity

To determine extracellular α-amylase activity and agarase activity, the supernatants from the aliquots of bacterial cell cultures were collected at the indicated growth phases and concentrated by precipitation with ammonium sulfate brought to 80% saturation; the precipitated protein was collected by centrifugation at 13 000*g* for 30 min and dissolved in 20 mM phosphate buffer (pH 7).

The activities of α-amylase and agarase were estimated by determining the amount of reducing sugars released from starch and agarose, respectively. α-amylase and agarase activities were determined as previously described [25,30]. One unit of α-amylase was defined as the amount of an enzyme necessary to produce reducing sugar equivalent to 1 µmol of glucose in 30 min under the assay conditions. The specific activity, measured as units per mg of protein, was the average of at least three independent determinations.

One unit of agarase activity is the amount of enzyme that increased absorbance at 450 nm by 0.001 per minute of incubation under the assay conditions. The specific activity was expressed as units per mg of dry weight and was the average of at least three independent determinations.

The FKBP activity was determined using protoplasts. Protoplasts were prepared as previously described [18]. Briefly, cells from 10 ml of NMMP culture were washed and re-suspended in P buffer according to the bacterial dry weight with 1 mg ml^−1^ lysozyme. After 20 min of incubation at 37°C protoplasts were washed with P buffer and then were centrifuged at 1500*g* for 5 min at 4°C. The protoplast's pellet was then re-suspended in 300 µl P buffer, and 30 µl aliquots were used to perform the assay.

The isomerase assay was determined by protease coupling assay with α-chymotrypsin (α-Ct) (Sigma Aldrich ref. C4129) using as a substrate *N*-succinyl-L-Ala-Ala-Pro-Phe-*p-*nitroanilide (sAAPF-pNA) (Sigma Aldrich ref. S7388). Chymotrypsin cleaves the synthetic peptide only when the Ala-Pro bond, which is in equilibrium between *cis* and *trans*, is in *trans* configuration [31]. The reaction was monitored for 6 min by the increase in absorbance at 360 nm (corresponding to the release of *p-*nitroanilide) with a spectrophotometer (Ultrospec 3100 pro; GE Healthcare, Amersham). FK-506 (Sigma Aldrich ref. F4679) was added in the inhibition of the PPIase activity assays [32]. PPIase catalyses the *cis/trans* isomerization of X-Pro peptide bonds. Therefore, the isomerization reaction of this peptide bond was measured by monitoring the release of *p*-nitroaniline.

Aliquots (30 µl) of the pIJ487 and pIJFKBP protoplasts were mixed with 625 µl of 110 mM Tris HCl pH 8 and water to a total volume of 710 µl and incubated for 15 min on ice. Then, 120 µl of α-Ct 0.56 mM dissolved in 1 mM HCl and 30 µl of sAAPF-pNA 1.4 mg ml^−1^ dissolved in dimethyl sulfoxide were added and incubated for 3 min on ice before the absorbance was measured at 390 nm for 1–6 min. For the inhibition assay 2 µl of FK506 14.2 µM dissolved in 50% (v/v) aqueous ethanol was added to the 30 µl aliquots and 625 µl of 110 mM Tris HCl pH 8 and then incubated for 15 min on ice. After that, chymotrypsin and sAAPF-pNA were added [32]. The residual activity was determined relative to a control sample treated identically in the absence of the inhibitor. All data are averaged from three independent measurements.

### 2.7. Protein predictions

For the prediction of the signal peptide SignalP 4.1, LipoP 1.0 and TatP 1.0 servers [33–35] were used. The presence of disulfide bonds in the proteins was predicted using the DIANNA 1.1 server [36]. The SPOT-Omega server was used to predict which proteins could contain potential *cis-trans* prolyl isomers [37].

### 2.8. Molecular modelling

Full three-dimensional protein models were generated using I-TASSER [38], Modeller [39], Raptor-X [40] and Sparks [41] by homology with known functional structures. After inspection, the best models were selected as potential representatives of the functional structure. Isomerized peptide bonds were identified using the VMD Cispeptide plugin [42,43]. Additional models with the peptide bonds reverted to the *trans* conformation were generated using UCSF Chimera [44] and subjected to additional refinement through a cycle comprising energy minimization, equilibration and 30 ns molecular dynamics (MD) in 150 mM saline solution using GROMACS [45] and the Amber [46] force field. All the selected models were then inspected to identify H-bonds and compute the charge distribution on their surface using UCSF Chimera and APBS [47].

### 2.9. Protein identification by nano-LC-MS/MS triple TOF analysis

For protein identification, the *S. lividans* (pAGAs5) (pFDTFKBP) extracellular protein band reacting positively with anti-agarase antibodies was sliced out of the SDS-PAGE and digested with trypsin using Proteineer DP robot (Bruker, Bremen, Germany). Digestion was performed according to a previously described protocol [48]. In summary, gel plugs were washed with 50 mM ammonium bicarbonate and samples reduced with 10 mM dithiothreitol. Alkylation was carried out with 55 mM iodoacetamide at room temperature before adding recombinant sequencing-grade trypsin (0.1 µg; Promega). Digestion took place at 37°C for 18 h. Following digestion, peptides were extracted, dried by speed-vac centrifugation and stored at −20°C until needed.

The peptide samples were analysed on a nano-liquid chromatography (LC) system (Eksigent Technologies nanoLC Ultra 1D plus; AB SCIEX, Foster City, CA) coupled to a 5600 TripleTOF mass spectrometer (AB SCIEX, Foster City, CA) with a nanoelectrospray ion source. Samples were loaded on a C18 PepMap trap column (5 µm particle size, 100 µm I.D. × 2 cm; Thermo Scientific) at 2 µl min^−1^, in 0.1% formic acid in water. The trap column was switched online to a C18 nanoAcquity BEH analytical column (1.7 µm, 100 Å, 75 µm I.D. ×15 cm; Waters). Chromatographic elution was achieved using a 40 min linear gradient ranging from 5% to 40% solvent B (0.1% formic acid in acetonitrile) at 250 nl min^−1^. The mass spectrometer operated in data-dependent acquisition mode. For TOF scans, the accumulation time was set to 250 ms, and 15 precursor ions were monitored per cycle.

Mass spectrometry (MS) and tandem mass spectrometry (MS/MS) spectra were processed using Analyst TF 1.5.1 software (AB SCIEX, Foster City, CA). Raw data were converted to MGFs (mascot general files) and searched against a database built from sequences obtained for *S. coelicolor* at Uniprot Knowledgebase (as of November 2016), using a licensed Mascot Server v. 2.4 search engine (Matrix Science, London, UK). Search parameters were set as follows: carbamidomethylation of cysteine as fixed modification and methionine oxidation as variable 1. Peptide mass tolerance was set to 25 ppm and 0.02 Da, in MS and MS/MS mode, respectively and one missed cleavage was allowed.

### 2.10. Targeted mass spectrometry (SRM/MRM)

Relative protein abundance of a selected (*n* = 36) group of proteins was determined by targeted proteomics, using selected reaction monitoring (SRM/MRM). For that purpose, 1 mg of trypsin-digested proteins per sample was analysed using an Eksigent 1D Plus nano-LC system coupled to a SCIEX 55000 QTRAP quadrupole triple mass spectrometer. Using a 45 min length gradient, 273 transitions that corresponded to 83 specific peptides of the 36 proteins of interest were monitored. Protein-specific peptides and transitions were selected, whenever possible, on the basis of previous experimental data. As the number of transitions was too high to analyse using a single SRM/MRM method, two sub-methods were designed. Nine samples (three conditions, three biological replicates per condition) were monitored using both sub-methods and the raw data files in WIFF format were analysed using the Skyline 4.2 software [49]. The analysis determined the areas corresponding to each transition and peptide. For those samples in which peptides could not be detected, quantification results were not considered. For those cases in which more than one peptide per protein was monitored, the areas corresponding to each protein-specific peptide were added to obtain a total summed area for each protein. Summed protein areas were used to calculate relative protein abundances between samples. The relative protein abundances were log_2_-transformed to approach normality and comparisons between different strains were performed using Student's *t*-test and Fisher's exact test.

## 3. Results

### 3.1. Identification of the *Streptomyces lividans* TK21 *fkbp* gene

Analysis of the *S. lividans* TK21 secretome identified a putative polypeptide similar to that of the *S. coelicolor* SCO1639 protein [27]. This gene encoded a putative Tat-dependent PPIase lipoprotein [50] that is orthologous to the previously characterized *Streptomyces chrysomallus* protein (FKBP-33) having a PPIase activity [51]. The *S. lividans* TK24 gene *SLIV_29545* has been shown to be highly homologous to the SCO1639.

To test if the equivalent *S. lividans* TK21 gene homologous to the *SLIV_29545* gene could also encode a PPIase with a potential role in the correct folding of secretory proteins, oligonucleotides FKBP-P and FKBP-T were used to amplify a 1222 bp long DNA fragment from the *S. lividans* genome containing the DNA sequence encoding the putative FKBP-like protein and the potential regulatory region of the gene.

The 1222 bp long amplified DNA fragment was sequenced and its sequence was aligned with the equivalent ones from the *S. coelicolor* and the *S. lividans* TK24 genomes and found to coincide in 99% and 100%, respectively, with that of the genes encoding their respective FKBP-like proteins. The FKBP-like protein encoded in *S. lividans* TK21 was predicted to be a substrate for a type II signal peptidase [34]. Moreover, the level of expression of the gene encoding the *S. lividans* TK21 FKBP-like protein was found to be lower in an *S. lividans* TK21 strain defective in the type II signal peptidase Lsp [52], when measured by qRT-PCR transcriptional analyses.

### 3.2. The putative *S. lividans* FKBP has PPIase activity

The *S. lividans* FKBP-like protein showed 36% identical residues and 50% equivalent amino acids to the *E. coli* periplasmic FKBP (FkpA), which acts as a periplasmic folding modulator with a broad substrate range [53]. Activity of FKBP-like PPIases is inhibited by the immunosuppressive compound FK506 [32].

**Figure 1.**
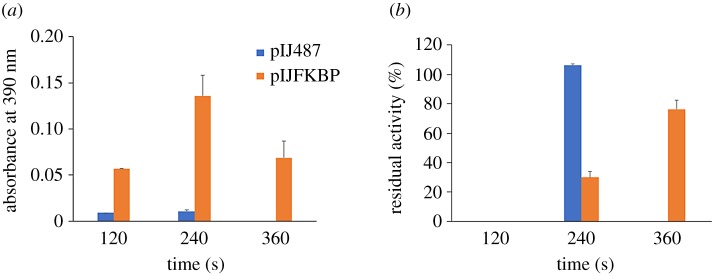
Assay of isomerase activity. *Streptomyces lividans* pIJ487 and *S. lividans* pIJFKBP protoplasts were tested for the PPIase activity and FK506 inhibition assays. (*a*) The isomerization activity was measured by monitoring the release of *p*-nitroaniline at 390 nm as indicated in the Methods section. (*b*) The inhibition assays were performed as indicated in the Methods section. The residual PPIase activity was calculated relative to the activity value of the sample treated identically in the absence of the inhibitor and expressed as a percentage.

Since *S. lividans* FKBP-like protein is a lipoprotein, to perform the PPIase activity assay we used protoplast extracts of the *S. lividans* pIJ486 and *S. lividans* pIJFKBP strains, containing the multi-copy plasmid pIJ486 and the multi-copy plasmid pIJ486 harbouring the *fkbp-*like gene, respectively (figure 1). The background values of the absorbance of the *p*-nitroaniline released from the substrate upon incubation in the absence of protoplasts were subtracted from those obtained in the presence of pIJ486 protoplasts and pIJFKBP protoplasts. A higher increase in the absorbance was observed for protoplasts containing pIJFKBP owing to the presence of an excess of FKBP copies versus the protoplast containing pIJ487 at 120 and 240 s (figure 1*a*).

The presence of the inhibitor FK506 decreases the absorbance to 0 at 120 s and recovers the absorbance observed with respect to the control sample (without inhibitor) at 240 s in the pIJ487 protoplasts, which represents a percentage of inhibition of 100% at 120 s. The values of residual activity after treatment with the inhibitor FK506 in the pIJFKBP protoplasts was 0%, 30% and 76.23%, which represent a percentage of inhibition of 100%, 70% and 23.77% at 120, 240 and 360 s, respectively (figure 1*b*)*.* From now on we will refer to the *S. lividans* TK21 gene encoding the FKBP-like protein as *sli-fkbp*.

### 3.3. A mutant in *sli-fkbp* induces the secretion stress response

The qRT-PCR transcriptional analyses, using as an endogenous control the *hrdB* gene, showed that the expression of the *sli-fkbp* and *tatC* in the *S. lividans* TK21 strain runs parallel to cell culture growth, although the level of the *sli-fkbp* expression is lower than that of the *tatC* gene (about 10% that of *tatC*) [26]. In the absence of *sli-fkbp* expression, the overall amount of misfolded secreted proteins would likely increase and, consequently, the level of expression of the two-component CssRS system [24,25] could be induced. Therefore, an *sli-fkbp* disrupted mutant (*sli-fkbp*::pOJ260) was constructed, and the relative level of expression of the CssRS two-component system genes was determined by qRT-PCR analyses and compared with that of the wild-type cell. A significant increase in the expression levels of the two genes (relative expression levels of the sensor *cssS*: 25.02 ± 1.35 and of the regulator *cssR*: 8.35 ± 1.11) was observed at the late exponential phase of growth (24 h), suggesting that the absence of the *sli-fkbp* gene may produce an accumulation of misfolded proteins at this time. This suggests that, despite the moderate level of expression of the single *sli-fkbp* copy present in the bacterial genome, the Sli-FKBP protein may play an active role in the correct folding of extracellular proteins.

### 3.4. The absence of *sli-fkbp* affects secretion and activity of Sec-dependent α-amylase

Prolyl isomerization to the *cis* conformation at residue 350 (P350) may be required to attain the mature form of the Sec-dependent protein α-amylase. Similarly, the Tat-dependent protein agarase may require prolyl isomerization at residues 127 and 183 to the *cis* conformation to produce mature active agarase (electronic supplementary material, figure S1).

To explore further the role of Sli-FKBP in the secretion of proteins in *S. lividans*, we propagated the multi-copy plasmids pAMI11 and pAGAs5 harbouring the *S. lividans* α-amylase gene (*amlB*) and the *S. coelicolor* agarase gene (*dagA*), respectively, in the *sli-fkbp* mutant and determined the secretion and extracellular activities of α-amylase and agarase.

**Figure 2.**
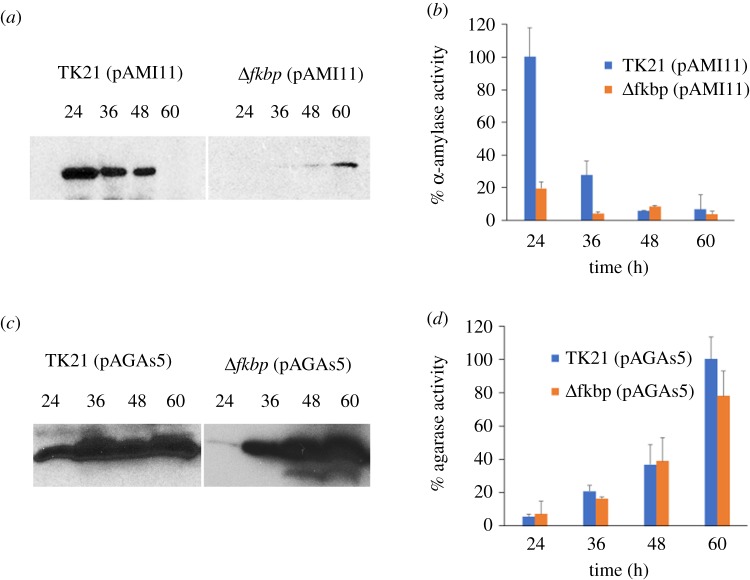
α-amylase and agarase secretion and extracellular activities in the *sli-fkbp* mutant. α-amylase secretion pattern (*a*), α-amylase activity (*b*), agarase secretion pattern (*c*) and agarase activity (*d*) were measured at different times of growth in the *sli-fkbp* mutant containing the plasmids pAMI11 (harbouring the α-amylase-encoding gene, *amlB*) or pAGAs5 (harbouring the agarase-encoding gene, *dagA*) propagated in multi-copy and compared with those of their respective wild-type counterparts. α-amylase and agarase secretion were analysed by western blotting with antibodies raised against AmlB (59 kDa) and DagA (32 kDa). The amount of protein loaded onto the gels was corrected by the dried weight of the bacterial cultures. Activities are expressed as percentages, where 100% is the maximum level of the α-amylase and agarase activities measured in the corresponding wild-type strain. Percentage values are the mean of the three different measurements.

Overexpression of *amlB* and *dagA* in the *sli-fkbp* mutant strain constrained their growth rate when compared with the wild-type (electronic supplementary material, figure S2a and S2b). The Sec-dependent protein α-amylase reaches its maximum level of secretion at 24 h of growth while agarase reaches its maximum level of secretion during the stationary phase [26] (figure 2*a*,*c*). The secretion pattern and extracellular activity of α-amylase were severely affected in the *sli-fkbp* mutant strain with respect to that of the isogenic strain (figure 2*a*,*b*), showing the presence of inactive α-amylase only at 60 h and probably corresponding to incorrectly folded protein as previously described [24,25]. In contrast, with the agarase, only moderate changes to the secretion pattern and extracellular activity were observed (figure 2*c*,*d*).

### 3.5. α-amylase and agarase activities increase when FKBP is overproduced

To study if overproduction of Sli-FKBP could increase the production of the Sec-dependent model protein α-amylase, the pFDTFKBP plasmid (a plasmid compatible with pIJ486) was propagated in the *S. lividans* TK21 (pAMI11) strain.

**Figure 3.**
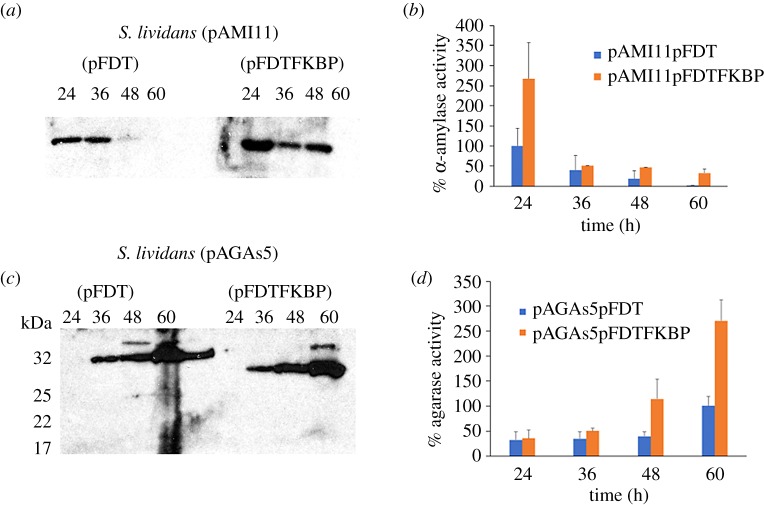
α-amylase and agarase secretion and extracellular activities in cells overproducing Sli-FKBP. The α-amylase secretion pattern (*a*), α-amylase activity (*b*), agarase secretion pattern (*c*) and agarase activity (*d*) were measured at different times of growth by propagation of the respective multi-copy plasmids pAMI11 or pAGAs5 concomitantly with the compatible multi-copy plasmids carrying the *sli-fkbp* gene. α-amylase and agarase secretion were analysed by western blotting with antibodies raised against AmlB (59 kDa) and DagA (32 kDa). The amount of protein loaded onto the gels was corrected by the dried weight of the bacterial cultures. Molecular size markers are indicated on the side of (*c*). Activities are expressed as percentages, where 100% is the maximal level of the α-amylase or agarase activities measured in the corresponding wild-type strain harbouring the compatible plasmid pFDT. Percentage values are the mean of three different measurements.

Overproduction of Sli-FKBP did not affect significantly the rate of growth in liquid medium (electronic supplementary material, figure S2c and S2d). α-amylase secretion increased, with an increase in extracellular activity of around threefold with respect to the isogenic strain overproducing α-amylase (figure 3*a*,*b*), suggesting that secreted α-amylase may benefit from the action of Sli-FKBP for correct folding. Moreover, the level of expression of the CssRS two-component system genes decreased in the strain that overproduces α-amylase and *sli-fkbp* (relative expression levels of the sensor: *cssS* −2.42 ± 0.113 and of the regulator *cssR*: −1.79 ± 0.89) versus the strain lacking multi-copy *sli-fkbp*, where the two-component system was activated [25]. This suggests that overproduction of some extracellular Sec-dependent proteins may be aided by the action of PPIases for the secretory proteins to acquire their correctly folded structure.

To test if higher levels of FKBP would be needed to improve the extracellular activity of secreted agarase, the multi-copy plasmid pFDTFKBP was propagated in the S*. lividans* (pAGAs5) strain. Extracellular agarase activity registered a threefold increase in the strain overexpressing agarase and *sli-fkbp* (figure 3*d*), suggesting that overproduction of Tat-dependent agarase needs additional activity of Sli-FKBP to fold correctly. No significant increase was detected in the amount of secreted agarase by western blot assays (figure 3*c*). Incidentally, the western blot analyses showed a difference between strains in the gel mobility of the protein produced (figure 3*c*). The faster moving band, corresponding to agarase and Sli-FKBP overproduction, was sliced out of the gel and the protein analysed by nano LC-MS/MS triple TOF MS. The proteomic analysis allowed recovery and identification of tryptic peptides of mature agarase, obtaining 65% coverage of the overall protein (electronic supplementary material, figure S3), which would correspond to 20 kDa according to ProtParam [54]. This size does not agree with the size of the protein deduced from its gel mobility. Gel mobility shifts in SDS-PAGE have been associated with changes in protein structure, as previously described [55,56], and might provide an explanation for the discordance between the sizes derived by proteomic analysis and SDS-PAGE.

### 3.6. Agarase modelling

The best agarase models were selected by inspecting the homology models obtained with various methods according to coherence with published structures and available biological information. MD simulations of the chosen models revealed the existence of one preferred conformation that was used as a representative for further analysis. The predicted structure contains two *cis* peptide bonds, at P127 and P183, equivalent to those found on other homologues. Switching of P127 to the *trans* conformation would disrupt the allosteric agarose-binding site, preserving the active site, while switching of P183 would disrupt both (electronic supplementary material, figure S4). The *trans* forms show a conformation with lower potential energy (more stability) than the *cis* structures. However, the mature double-*cis* form contains numerous H-bonds that help stabilize it. This suggests that the spontaneous change from *trans* to *cis* would be energetically unfavourable, justifying the need for a PPIase to facilitate it, while the H-bond network in the *cis* form would provide a significant initial barrier to spontaneous reversion to the *trans* conformation afterwards.

In addition, the *trans* forms would have larger molecular dimensions (as evidenced by a larger radius of gyration, equivalent to the Stokes radius), and the distribution of surface charges would also be different: conversion to *cis* would be associated with a larger reduction (greater than 400 Å^2^) of surface positive charge than of the surface negative charge. Both of these effects (changes in dimension and surface charge) could contribute to the differential mobility of the *cis* and *trans* forms.

### 3.7. Effect of Sli-FKBP on secretory proteins

To gain further insight into the effect of the overproduction of Sli-FKBP on secreted proteins we performed an SRM/MRM proteomic experiment. SRM/MRM is typically used to perform reliable, sensitive and selective protein assays for the comparison of relative protein amounts between samples [57]. The analyses were performed using supernatants from early stationary phase cultures of the Δ*sli-fkbp*, the single-copy *sli-fkbp* and the *sli-fkbp* overexpressing strains using three biological replicates.

Cognate secreted proteins previously identified in the supernatant of *S. lividans* TK21 were analysed with the SPOT-Omega server [37] to identify those with a high probability of containing proline *cis–trans* isomers. A group of 31 secreted proteins that might be potential substrates of Sli-FKBP (electronic supplementary material, table S1) were selected for further analysis using SRM/MRM. Sli-FKBP was also included in the analysis to compare its expression in the three strains.

None of the peptides corresponding to four of the secreted proteins analysed could be detected in any of the strains (electronic supplementary material, table S1) and these proteins were not considered in the analysis, leaving 27 proteins. Twelve proteins had undetected peptides in the Δ*sli-fkbp* strain but were detected in the other strains. The ratios and significance level (*p*-value < 0.05) of differences in relative protein abundances were calculated for all proteins detected. The results are summarized in table 1.

**Table 1.** Secreted proteins significantly affected by Sli-FKBP. Protein function was annotated according to StoPSserver [7]. ∅︀ indicates that no peptide was detected in any of the replicates. Ratio: ratio of protein abundances. *p*: *p*-value. NC: not computable (division by zero). Analyses were performed using three biological replicates. Only statistically significant values (*p* < 0.05) are reported to avoid reporting potentially misleading results. Post-translocational modification proteins are shown in italics.

gene	annotated function	route	relative protein abundance			pFDT-FKBP versus pFDT		pFDT-FKBP versus Δ*fkbp*	
			pFDT-FKBP	pFDT	Δ*fkbp*	ratio	*p*	ratio	*p*
SLI01600	glycoside hydrolase, family 43	Tat	29928	15411	∅︀			NC	0.0004
SLI03045	*N*-acetylmuramoyl-L-alanine amidase	Sec	57863	24139	∅︀	2.4	0.045	NC	0.001
SLI03130	alpha/beta hydrolase fold protein	Sec	116228	63987	∅︀			NC	7 × 10^−5^
SLI03390	amidase	Tat	59108	9263	713			82.9	0.023
SLI04830	metallopeptidase	Sec	202438	247559	8906	0.8	0.004	22.7	4 × 10^−5^
SLI04955	soluble quinoprotein glucose/sorbosone dehydrogenase	Sec	298908	145641	891	2.1	0.022	335.4	0.018
SLI06140	beta-galactosidase (lactase)	Tat	29931	19031	653			45.8	0.010
SLI07140	esterase	Sec	5483255	4583879	186258			29.4	0.006
SLI07150	uncharacterized protein	Tat	207033	132261	12380			16.7	0.018
SLI07590	peptidase S1A, chymotrypsin family	Sec	4485423	3569046	200789	1.3	0.047	22.3	0.009
SLI08115	alpha-1,2-mannosidase, putative	Sec	209808	72118	∅︀			NC	0.001
SLI09630	peptidase S1, PA clan	Tat	49908	52465	∅︀			NC	2 × 10^−4^
SLI10325	peptidase	Sec	56001	33131	7483	1.7	0.049	7.4	0.001
SLI12065	solute-binding protein	Sec	36343	48931	1692			21.4	0.009
SLI14975	secreted endopeptidase, NLPC/P60 domain	Tat	204846	92338	1514			135.3	0.010
SLI19350	protein of unknown function containing DUF839 domain	Tat	28591	14074	∅︀	2.0	0.020	NC	0.001
SLI19735	glycoside hydrolase/deacetylase	Sec	517430	458383	12514			41.3	0.002
SLI22430	esterase	Sec	10354280	8344363	39145			264.5	0.004
SLI23095	peptidase M6, InhA family protein	Tat	424058	345723	∅︀			NC	0.001
SLI25270	unknown function	Sec	33439	31383	∅︀	1.1	0.047	NC	1 × 10^−5^
SLI25665	aldose 1-epimerase	Tat	32022	12157	∅︀	2.6	0.027	NC	0.001
SLI26155	*N*-acetylmuramoyl-L-alanine amidase	Tat	481504	266240	24205			19.8	0.003
SLI27125	*N*-acetylmuramoyl-L-alanine amidase domain with Tachylectin 2	Sec	716759	634345	10642			67.3	0.010
SLI29775	glycosyl hydrolase-like 10 (UPFUPF0748-like protein)	Tat	182360	262400	∅︀			NC	4 × 10^−5^
SLI31645	tripeptidyl aminopeptidase	Sec	70306	47246	∅︀			NC	0.001
SLI34120	probable subtilase-type protease inhibitor	Sec	1457618	1424560	376745			3.8	0.030
SLI34825	glycosyl hydrolase, five-bladed beta-propeller domain	Tat	75741	97855	∅︀			NC	1 × 10^−4^
SLI29545	PPIase	Tat	636700	372348	5509	1.7	0.026	115.5	0.020
*SLI18490*	*putative copper chaperone SCO1/SenC*	*Sec*	*2028918*	*892015*	*26378*	*2*.*275*	*0*.*005*	*76*.*9*	*1 × 10^−4^*
*SLI26855*	*HtrA1 protease*	*Sec*	*99782*	*88957*	*28550*			*3*.*4*	*0*.*005*

As expected, Sli-FKBP (SLI29545) showed significant differences in the relative protein abundance among the three strains according to their corresponding genotype. Comparison of the extreme cases pFDTFKBP and Δ*sli-fkbp* strains should highlight the effect of FKBP. All the proteins (both Sec and Tat secreted) were significantly affected in the Δ*sli-fkbp* strain with respect to the overproducing strain; the relative expression ratio could not be calculated for proteins not detected in the Δ*sli-fkbp* strain. On the other hand, comparison of pFDTFKBP with the isogenic pFDT strain should tag proteins that may be affected by overexpression of *sli-fkbp*. Besides the expected difference in Sli-FKBP, nine other secretory proteins displayed a significant difference in their relative abundance when Sli-FKBP was expressed in multi-copy (pFDTFKBP) versus single-copy (pFDT) strains.

The increments in relative protein abundances observed in the presence of Sli-FKBP suggest that it may have a role in contributing to improve the stability of a wide number of proteins.

The stabilizing effect of Sli-FKBP might be indirect, owing to isomerization of intermediate proteins with post-translocational activity over the final secretory proteins. For this reason, we also included in the MRM/SRM experiment two thiol-disulfide oxidoreductases recently described in *S. lividans* TK21, Sli-DsbA and Sli-DsbC [58], a protease that degrades misfolded proteins and may have a chaperone-like role, HtrA1 (SLI26855) [24], and the only extracellular chaperone detected to date in the secretome of *S. lividans* (SLI18490) [59]. No peptides were detected for the two thiol-disulfide oxidoreductases (electronic supplementary material, table S1). We detected a significant increase in the relative abundance of chaperone (SLI18490) between the Sli-FKBP overexpressing strain pFDTFKBP and the isogenic pFDT strain (table 1), but not for protease HtrA1 (SLI26855).

## 4. Discussion

PPIases have been found to be present in almost all sequenced genomes and to be involved in a variety of biological processes [53,60–65]. Recently, the *S. lividans* TK24 secretome [59] has been reported showing only one extracytoplasmic PPIase in *S. lividans*, SLIV_29545, which is homologous to SCO1639. The *S. coelicolor* FKBP-like SCO1639 has been identified as a Tat-dependent lipoprotein [50], but little is known about its function in the folding of secretory proteins. We have identified and characterized an FKBP-like homologue in *S. lividans* TK21 and shown that this protein (which we have named Sli-FKBP) has a PPIase activity that can be inhibited by FK506 (figure 1*a*,*b*).

The accumulation of secretory misfolded proteins induces a secretion stress response that activates the two-component system, CssRS, which regulates the synthesis of three specific HtrA-like proteases able to degrade misfolded proteins at the late exponential growth phase [25]. As expected, the disruption of the *sli-fkbp* gene increased the transcriptional level of the CssRS operon, suggesting accumulation of misfolded proteins. In addition, absence of *sli-fkbp* severely affects secretion and activity of Sec-dependent α-amylase (figure 2*a*,*b*), similarly to what has been reported with the *prsA* mutant [14,16,66,67]. Furthermore, absence of *sli-fkbp* moderately affects the secretion and activity of Tat-dependent agarase (figure 2*c*,*d*), a protein previously thought to be secreted in a fully folded conformation.

Accordingly, prolyl isomerization to the *cis* conformation may be involved in Sec-dependent α-amylase (at position P350) and Tat-dependent agarase (at positions P127 and P183) (electronic supplementary material, figure S1) to render the mature active enzymes. Further research is needed to study the implication of Sli-FKBP and these proline residues in their folding and activity.

When the *sli-fkbp* gene was propagated in multi-copy in the α-amylase overproducer strain, secretion and activity increased (figure 3*a*,*b*). It has been described that overproduction of PrsA enhances the secretion of α-amylase from *Bacillus amyloliquefaciens* (AmyQ) [14], affecting its folding. Taken together with our results, this suggests that activity of an FKBP-like PPIase may be important for the acquisition of their respective active conformation in the case of some Sec-dependent secreted proteins. In addition, it strongly suggests that bacterial cells may need extra copies of this folding enzyme when secretory proteins are overproduced.

In the case of α-amylase overproduction, which is expected to form five disulfide bonds, maturation may involve the participation of thiol-disulfide oxidoreductases, as recently published [58], in addition to increased levels of the PPIase.

Despite the Sli-FKBP lipoprotein being predicted to contain a Tat signal peptide [34] and although it has been described that the Tat route is mainly expressed at the late phase of growth [26], the presence of Sli-FKBP has been detected in the secretome of *S. lividans* TK21 around the end of the exponential phase of growth [27], similar to findings reported for the *S. lividans* TK24 secretome [59]. Tat-dependent agarase shows a similar behaviour, and it has additionally been found that, under overproduction, pre-agarase may be partially targeted to the Sec route and be secreted unfolded (in contrast to the proposed folded conformation produced through the Tat route) [8]. The presence of Sli-FKBP in the secretome to affect Sec-dependent proteins earlier than what would otherwise be expected for a typical Tat protein might be explained by a similar mechanism.

Overproduction of Sli-FKBP increased activity of agarase in a proportion greater than what would be expected only by secretion (figure 3*c*,*d*). Remarkably, under agarase overproduction conditions, we also observed a shift in its SDS-PAGE mobility that only occurred when Sli-FKBP was overproduced concomitantly (figure 3*b*). Analysis of the peptides produced by trypsin digestion of the protein recovered from the faster migrating band reported identification of most of the agarase protein sequence (electronic supplementary material, figure S3). The observed band migration does not correspond to the mobility that would be expected if the lack of detection of unrecovered peptides had been due to specific protein degradation. Furthermore, specific protein degradation would conflict with the higher levels of agarase activity measured. Thus, the difference in mobility can hardly be explained just by protein degradation, and seems more likely to be due to gel shifting, a phenomenon previously described where mobility changes in SDS-PAGE gels are explained by changes in the protein conformation that affect its Stokes radius or surface charge. Actually, inspection of the structural models produced for agarase agrees with this observation.

The increase in agarase activity when Sli-FKBP is concomitantly overproduced might be justified by differences in the degree of agarase folding assisted by Sli-FKBP. According to modelling, the form P183*cis*, P127*trans* might keep an active catalytic site without an allosteric agarose-binding site, lacking processive ability with reduced efficiency. This could explain why, in unsaturated Sli-FKBP conditions, agarase overproduction may display lower activity than with Sli-FKBP overproduction (electronic supplementary material, figure S4).

The dependency of agarase activity on Sli-FKBP overproduction may provide an interesting model for introducing improvements to the industrial overproduction of other Sli-FKBP-dependent secreted proteins.

In any case, a relevant proportion of the proteins analysed by SRM/MRM showed a dependence of their relative abundance on Sli-FKBP when the Δ*sli-fkbp* strain was used or when *sli-fkbp* was overexpressed, indicating that Sli-FKBP may have a wide relevance for protein secretion (table 1).

This is especially striking in the case of proteins secreted through the Tat route. Tat-dependent proteins are very few in comparison with Sec-dependent ones; not all of the verified 27 Tat-dependent proteins [10] may be produced in experimental growth conditions, and they are thought to be exported fully folded having a relatively simple tertiary structure, containing few or no disulfide bonds [68]. Tat-dependent secretory proteins containing *cis* bonds could reach their mature form either spontaneously or with the assistance of cytoplasmic PPIases [59]. In a situation of overproduction, the isomerization step could be limited, so the overproduction of an Sli-FKBP may help extracellularly to form more active protein in the periplasm. In the case of agarase, which lacks disulfide bonds, the thiol-disulfide oxidoreductase lipoprotein Sli-DsbA may also act as a chaperone to help obtain mature agarase [58]. Unfortunately, the MRM/SRM analyses failed to detect Sli-DsbA, but could detect the extracellular protein SLI18490, the single annotated chaperone in the secretome of *S. lividans* [59]. SLI18490 is also a lipoprotein, and its relative protein abundance increases in the presence of Sli-FKBP. The possible relationship between Sli-FKBP and SLI18490 in *S. lividans* could be related to the morphological differentiation as in other streptomycetes [63,69]. Together, Sli-FKBP and other chaperones (such as Sli-DsbA) could assist the folding of overproduced secreted proteins in *S. lividans* in a similar way to *E. coli*, where two periplasmic folding factors (chaperone Skp and PPIase FkpA) are involved in protein folding in the periplasm [53]. Further experimental work is required to characterize the actual relationships between Sli-FKBP, Sli-DsbA and SLI18490.

## 5. Conclusion

We have identified an FKBP-like protein in *S. lividans* that may be involved in the correct folding of a wide number of Sec- and Tat-dependent secretory proteins. Our results indicate that, in practical terms and regardless of the route used, when the relative amount of overproduced secretory protein exceeds the capacity of the proteins in charge of procuring them a correctly folded structure, it may be convenient to adjust the level of expression of specific folding enzymes to ensure complete folding and optimize the overproduction of functional homologous or heterologous secretory proteins in *S. lividans*.

## Supplementary Material

Figure S1. Alpha-amylase and agarase models.; Figure S2. Growth curves of the sli-fkbp mutant and Sli-FKBP overproducer strains overexpressing amlB and dagA.; Figure S3. Agarase sequence coverage obtained by nano LC-MS/MS Triple TOF analysis.; Figure S4. Predicted models of agarase.

Reviewer comments

## Supplementary Material

Table S1

## Data accessibility

This article has no additional data.

## References

[1] Gilbert M, Morosoli R, Shareck F, Kluepfel D 1995 Production and secretion of proteins by streptomycetes. Crit. Rev. Biotechnol. 15, 13–19. (10.3109/07388559509150530) 7736599

[2] Chater KF 1998 Taking a genetic scalpel to the *Streptomyces* colony. Microbiology 144, 1465–1478. (10.1099/00221287-144-6-1465) 33789395

[3] Cruz-Morales P et al. 2013 The genome sequence of *Streptomyces lividans* 66 reveals a novel tRNA-dependent peptide biosynthetic system within a metal-related genomic island. Genome Biol. Evol. 5, 1165–1175. (10.1093/gbe/evt082) 23709624 PMC3698927

[4] Rückert C et al. 2015 Complete genome sequence of *Streptomyces lividans* TK24. J. Biotechnol. 199, 21–22. (10.1016/j.jbiotec.2015.02.004) 25680930

[5] Vrancken K, Anné J 2009 Secretory production of recombinant proteins by *Streptomyces**.* Future Microbiol. 4, 181–188. (10.2217/17460913.4.2.181) 19257845

[6] Anné J, Maldonado B, Van Impe J, Van Mellaert L, Bernaerts K. 2012 Recombinant protein production and streptomycetes. J. Biotechnol. 158, 159–167. (10.1016/j.jbiotec.2011.06.028) 21777629

[7] Tsolis KC et al. 2018 Comprehensive subcellular topologies of polypeptides in *Streptomyces*. Microb. Cell Fact. 17, 43 (10.1186/s12934-018-0892-0) 29544487 PMC5853079

[8] Palacín A, de la Fuente R, Valle I, Rivas LA, Mellado RP. 2003 *Streptomyces lividans* contains a minimal functional signal recognition particle that is involved in protein secretion. Microbiology 149, 2435–2442. (10.1099/mic.0.26313-0) 12949169

[9] Robinson C, Bolhuis A 2004 Tat-dependent protein targeting in prokaryotes and chloroplasts. Biochim. Biophys. Acta 1694, 135–147. (10.1016/j.bbamcr.2004.03.010) 15546663

[10] Widdick DA, Dilks K, Chandra G, Bottrill A, Naldrett M, Pohlschroder M, Palmer T 2006 The twin-arginine translocation pathway is a major route of protein export in *Streptomyces coelicolor*. Proc. Natl Acad. Sci. USA 103, 17 927–17 932. (10.1073/pnas.0607025103) PMC1693849 17093047

[11] De Keersmaeker S, Vrancken K, Van Mellaert L, Anné J, Geukens N. 2007 The tat pathway in *Streptomyces lividans*: interaction of Tat subunits and their role in translocation. Microbiology 153, 1087–1094. (10.1099/mic.0.2006/003053-0) 17379717

[12] Palmer T, Berks BC 2012 The twin-arginine translocation (Tat) protein export pathway. Nat. Rev. Microbiol. 11, 483–496. (10.1038/nrmicro2814) 22683878

[13] Göthel SF, Marahiel MA 1999 Peptidyl-prolyl cis-trans isomerases, a superfamily of ubiquitous folding catalysts. Cell. Mol. Life Sci. 55, 423–436. (10.1007/s000180050299) 10228556 PMC11146858

[14] Vitikainen V, Pummi T, Airaksinen U, Wahlström E, Wu H, Sarvas M, Kontinen VP 2001 Quantitation of the capacity of the secretion apparatus and requirement for PrsA in growth and secretion of alpha-amylase in *Bacillus subtilis*. J. Bacteriol. 183, 1881–1890. (10.1128/JB.183.6.1881-1890.2001) 11222585 PMC95082

[15] Vitikainen M et al 2004 Structure-function analysis of PrsA reveals roles for the parvulin-like and flanking N- and C-terminal domains in protein folding and secretion in *Bacillus subtilis**.* J. Biol. Chem. 279, 19 303–19 314. (10.1074/jbc.M400861200) 14976191

[16] Kontinen VP, Sarvas M 1993 The PrsA lipoprotein is essential for protein secretion in *Bacillus subtilis* and sets a limit for high-level secretion. Mol. Microbiol. 8, 727–737. (10.1111/j.1365-2958.1993.tb01616.x) 8332065

[17] Hopwood DA et al. 1985 Genetic manipulation of Streptomyces. A laboratory manual, p. 356 Norwich, UK: The John Innes Foundation and Cold Spring Harbour laboratory.

[18] Kieser T, Bibb MJ, Buttner MJ, Chater KF, Hopwood DA 2000 Practical Streptomyces genetics. Norwich, UK: John Innes Foundation.

[19] Bierman M, Logan R, O'Brien K, Seno ET, Rao RN, Schoner BE 1992 Plasmid cloning vector for conjugal transfer of DNA from *Escherichia coli* to *Streptomyces* spp. Gene 116, 43–49. (10.1016/0378-1119(92)90627-2) 1628843

[20] Nybo SE, Shepherd MD, Bosserman MA, Rohr J 2010 Genetic manipulation of *Streptomyces* species. Curr. Protoc. Microbiol. 19, 10E.3.1–10E.3.26. (10.1002/9780471729259.mc10e03s19) PMC11328516 21053253

[21] Flett F, Mersinias V, Smith CP 1997 High efficiency intergeneric conjugal transfer of plasmid DNA from *Escherichia coli* to methyl DNA-restricting streptomycetes. FEMS Microbiol. Lett. 155, 223–229. (10.1111/j.1574-6968.1997.tb13882.x) 9351205

[22] Palomino C, Mellado RP 2008 Influence of a *Streptomyces lividans* SecG functional analogue on protein secretion. Int. Microbiol. 11, 25–31. 18683629

[23] Palacín A, Parro V, Geukens N, Anné J, Mellado RP 2002 SipY is the *Streptomyces lividans* type I signal peptidase exerting a major effect on protein secretion. J. Bacteriol. 184, 4875–4880. (10.1128/JB.184.17.4875-4880.2002) 12169613 PMC135301

[24] Vicente RL, Gullón S, Marín S, Mellado RP 2016 The three *Streptomyces lividans* HtrA-like proteases involved in the secretion stress response act in a cooperative manner. PLoS ONE 11, e0168112 (10.1371/journal.pone.0168112) 27977736 PMC5157995

[25] Gullón S, Vicente RL, Mellado RP 2012 A novel two-component system involved in secretion stress response in *Streptomyces lividans*. PLoS ONE 7, e48987 (10.1371/journal.pone.0048987) 23155440 PMC3498368

[26] Gullón S, Marín S, Mellado RP 2015 Overproduction of a model Sec- and Tat-dependent secretory protein elicits different cellular responses in *Streptomyces lividans*. PLoS ONE 10, e0133645 (10.1371/journal.pone.0133645) 26200356 PMC4511581

[27] Escutia MR, Val G, Palacín A, Geukens N, Anné J, Mellado RP 2006 Compensatory effect of the minor *Streptomyces lividans* type I signal peptidases on the SipY major signal peptidase deficiency as determined by extracellular proteome analysis. Proteomics 6, 4137–4146. (10.1002/pmic.200500927) 16786486

[28] Laemmli UK 1970 Cleavage of structural proteins during the assembly of the head of bacteriophage T4. Nature 227, 680–685. (10.1038/227680a0) 5432063

[29] Timmons TM, Dunbar BS 1990 Protein blotting and immunodetection. Methods Enzymol. 182, 679–688 (10.1016/0076-6879(90)82053-5) 2314263

[30] Parro V, Mellado RP 1994 Effect of glucose on agarase overproduction by *Streptomyces*. Gene 145, 49–55. (10.1016/0378-1119(94)90321-2) 8045423

[31] Fischer G, Bang H, Berger E, Schellenberger A 1984 Conformational specificity of chymotrypsin toward proline-containing substrates. Biochim. Biophys. Acta 791, 87–97. (10.1016/0167-4838(84)90285-1) 6498206

[32] Zarnt T, Lang K, Burtscher H, Fischer G 1995 Time-dependent inhibition of peptidylprolyl *cis-trans*-isomerases by FK506 is probably due to *cis-trans* isomerization of the inhibitor's imide bond. Biochem. J. 305, 159–164. (10.1042/bj3050159) 7529995 PMC1136444

[33] Nielsen H 2017 Predicting secretory proteins with Signal P. Methods Mol. Biol. 1611, 59–73. (10.1007/978-1-4939-7015-5_6) 28451972

[34] Rahman O, Cummnigs SP, Harrington DJ, Sutcliffe IC 2008 Methods for the bioinformatics identification of bacterial lipoproteins encoded in the genome of Gram-positive bacteria. World J. Microbiol. Biotechnol. 24, 2377–2382. (10.1007/s11274-008-9795-2)

[35] Bendtsen JD, Nielsen H, Widdick D, Palmer T, Brunak S 2005 Prediction of twin-arginine signal peptides. BMC Bioinformatics 6, 167 (10.1186/1471-2105-6-167) 15992409 PMC1182353

[36] Ferrè F, Clote P 2006 DIANNA 1.1: an extension of the DIANNA web server for ternary cysteine classification. Nucleic Acids Res. 34, W182–W185. (10.1093/nar/gkl189) 16844987 PMC1538812

[37] Singh J, Hanson J, Heffernan R, Paliwal K, Yang Y, Zhou Y 2018 Detecting proline and non-proline cis isomers in protein structures from sequences using deep residual ensemble learning. J. Chem. Inf. Model. 58, 2033–2042. (10.1021/acs.jcim.8b00442). 30118602

[38] Zhang Y 2008 I-TASSER server for protein 3D structure prediction. BMC Bioinformatics 9, 40 (10.1186/1471-2105-9-40) 18215316 PMC2245901

[39] Eswar N, Webb B, Marti-Renom MA, Madhusudhan MS, Eramian D, Shen MY, Pieper U, Sali A 2006 Comparative protein structure modeling using Modeller. Curr. Protoc. Bioinformatics 15, 5.6.1–5.6.30. (10.1002/0471250953.bi0506s15) PMC4186674 18428767

[40] Källberg M, Wang H, Wang S, Peng J, Wang Z, Lu H, Xu J 2012 Template-based protein structure modeling using the RaptorX web server. Nat. Protoc 7, 1511–1522. (10.1038/nprot.2012.085) 22814390 PMC4730388

[41] Yang Y, Faraggi E, Zhao H, Zhou Y 2011 Improving protein fold recognition and template-based modeling by employing probabilistic-based matching between predicted one-dimensional structural properties of the query and corresponding native properties of templates. Bioinformatics 27, 2076–2082. (10.1093/bioinformatics/btr350) 21666270 PMC3137224

[42] Humphrey W, Dalke A, Schulten K 1996 VMD: visual molecular dynamics. J. Mol. Graph. 14, 33–38. (10.1016/0263-7855(96)00018-5) 8744570

[43] Schreiner E, Trabuco LG, Freddolino PL, Schulten K 2011 Stereochemical errors and their implications for molecular dynamics simulations. BMC Bioinformatics 12, 190 (10.1186/1471-2105-12-190) 21605430 PMC3124434

[44] Pettersen EF, Goddard TD, Huang CC, Couch GS, Greenblatt DM, Meng EC, Ferrin TE 2004 UCSF Chimera—a visualization system for exploratory research and analysis. J. Comput. Chem. 25, 1605–1612. (10.1002/jcc.20084) 15264254

[45] Abraham MJ, Murtola T, Schulz R, Páll S, Smith JC, Hess B, Lindahl E 2015 GROMACS: high performance molecular simulations through multi-level parallelism from laptops to supercomputers. SoftwareX 1–2, 19–25. (10.1016/j.softx.2015.06.001)

[46] Lindorff-Larsen K, Piana S, Palmo K, Maragakis P, Klepeis JL, Dror RO, Shaw DE 2010 Improved side-chain torsion potentials for the Amber ff99SB protein force field. Proteins 78, 1950–1958. (10.1002/prot22711) 20408171 PMC2970904

[47] Zhou YC, Feig M, Wei GW 2008 Highly accurate biomolecular electrostatics in continuum dielectric environments. J. Comput. Chem. 29, 87–97. (10.1002/jcc.20769) 17508411

[48] Schevchenko A, Wilm M, Vorm O, Mann M 1996 Mass spectrometric sequencing of proteins from silver stained polyacrylamide gels. Anal. Chem. 68, 850–858. (10.1021/ac950914h) 8779443

[49] MacLean B et al. 2010 Skyline: an open source document editor for creating and analyzing targeted proteomics experiments. Bioinformatics 26, 966–968. (10.1093/bioinformatics/btq054) 20147306 PMC2844992

[50] Thompson BJ, Widdick DA, Hicks MG, Chandra G, Sutcliffe IC, Palmer T, Hutchings MI 2010 Investigating lipoproteins biogenesis and function in the model Gram-positive bacterium *Streptomyces coelicolor*. Mol. Microbiol. 77, 943–957. (10.1111/j.1365-2958.2010.07261.x) 20572939

[51] Pahl A, Keller U 1994 *Streptomyces chrysomallus* FKBP-33 is a novel immunophilin consisting of two FK506 binding domains; its gene is transcriptionally coupled to the FKBP-12 gene. EMBO J. 13, 3472–3480. (10.1002/j.1460-2075.1994.tb06653.x) 8062824 PMC395250

[52] Gullón S, Arranz EIG, Mellado RP 2013 Transcriptional characterisation of the negative effect exerted by a deficiency in type II signal peptidase on extracellular protein secretion in *Streptomyces lividans**.* Appl. Microbiol. Biotechnol. 97, 10 069–10 080. (10.1007/s00253-013-5219-9) 24068336

[53] Baneyx F, Mujacic M 2004 Recombinant protein folding and misfolding in *Escherichia coli*. Nat. Biotechnol. 22, 1399–1408. (10.1038/nbt1029) 15529165

[54] Gasteiger E, Garriker A, Hoogland C, Ivanyi I, Appel RD, Bairoch A 2003 ExPASy: the proteomics server for in-depth protein knowledge and analysis. Nucleic Acids Res. 31, 3784–3788. (10.1093/nar/gkg563) 12824418 PMC168970

[55] Rath A, Glibowicka M, Nadeau VG, Chen G, Deber CM 2009 Detergent binding explains anomalous SDS-PAGE migration of membrane proteins. Proc. Natl Acad. Sci. USA 106, 1760–1765. (10.1073/pnas.0813167106) 19181854 PMC2644111

[56] Shi Y, Mowery RA, Ashley J, Hentz M, Ramirez AJ, Bilgicer B, Slunt-Brown H, Borchelt DR, Shaw BF 2012 Abnormal SDS-PAGE migration of cytosolic proteins can identify domains and mechanisms that control surfactant binding. Protein Sci. 21, 1197–1209. (10.1002/pro.2107) 22692797 PMC3537240

[57] Vidova V, Spacil Z 2017 A review on mass spectrometry-based quantitative proteomics: targeted and data independent acquisition. Anal. Chim. Acta 964, 7–23. (10.1016/j.aca.2017.01.059) 28351641

[58] Gullón S, Marín S, Mellado RP 2019 Four thiol-oxidoreductases involved in the formation of disulphide bonds in the *Streptomyces lividans* TK21 secretory proteins. Microb. Cell Fact. 18, 126 (10.1186/s12934-019-1175-0) 31345224 PMC6657201

[59] Tsolis KC et al 2019 Secretome dynamics in a Gram positive bacterial model. Mol. Cell. Proteomics. 18, 423–436. (10.1074/mcp.RA118.000899) 30498012 PMC6398212

[60] Hanes SD 2015 Prolyl isomerases in gene transcription. Biochim. Biophys. Acta 1850, 2017–2034. (10.1016/j.bbagen.2014.10.028) 25450176 PMC4417086

[61] Thapar R 2015 Roles of prolyl isomerases in RNA-mediated gene expression. Biomolecules 5, 974–999. (10.3390/biom5020974) 25992900 PMC4496705

[62] Sarvas M, Harwood CR, Bron S, van Dijl JM 2004 Post-translocational folding of secretory proteins in Gram-positive bacteria. Biochim. Biophys. Acta 1694, 311–327. 15546674 10.1016/j.bbamcr.2004.04.009

[63] Strakova E, Bobek J, Zikova A, Rehulka P, Benada O, Rehulkova H, Kofronova O, Vohradsky J 2013 Systems insight into the spore germination of *Streptomyces coelicolor*. J. Proteome Res. 12, 525–536. (10.1021/pr300980v) 23181467

[64] Kromina KA, Ignatov AN, Abdeeva IA 2008 Role of peptidyl-prolyl-*cis/trans*-isomerases in pathologic processes. Biochem. Moscow Suppl. Ser. A 2, 195 (10.1134/S199074780803001X)

[65] Ünal CM, Steinert M 2014 Microbial peptidyl-prolyl cis/trans isomerases (PPIases): virulence factors and potential alternative drug targets. Microbiol. Mol. Biol. Rev. 78, 544–571. (10.1128/MMBR.00015-14) 25184565 PMC4187684

[66] Jacobs M, Andersen JB, Kontinen V, Sarvas M 1993 *Bacillus subtilis* PrsA is required *in vivo* as an extracytoplasmic chaperone for secretion of active enzymes synthesized either with or without pro-sequences. Mol. Microbiol. 8, 957–966. (10.1111/j.1365-2958.1993.tb01640.x) 8102773

[67] Leskelä S, Wahlström E, Kontinen VP, Sarvas M 1999 Lipid modification of prelipoproteins is dispensable for growth but essential for efficient protein secretion in *Bacillus subtilis*: characterization of the *lgt* gene. Mol. Microbiol. 31, 1075–1085. (10.1046/j.1365-2958.1999.01247.x) 10096076

[68] Gullón S, Vicente RL, Valverde JR, Marín S, Mellado RP 2015 Exploring the feasibility of the Sec route to secrete proteins using the Tat route in *Streptomyces lividans*. Mol. Biotechnol. 57, 931–938. (10.1007/s12033-015-9883-0) 26202494

[69] Fujimoto M, Yamada A, Kurosawa J, Kawata A, Beppu T, Takano H, Ueda K 2012 Pleiotropic role of the Sco1/SenC family copper chaperone in the physiology of *Streptomyces**.* Microb. Biotechnol. 5, 477–488. (10.1111/j.1751-7915.2011.00319.x) 22117562 PMC3815325

