# Supplementary material for: Functional identification of a Streptomyces lividans FKBP-like protein involved in the folding of overproduced secreted proteins
Source: Open Biol. 2019 Oct 30;9(10):190201. doi: 10.1098/rsob.190201 (PMC6833217; doi:10.1098/rsob.190201)
Supplement: Figure S1. Alpha-amylase and agarase models.; Figure S2. Growth curves of the sli-fkbp mutant and Sli-FKBP overproducer strains overexpressing amlB and dagA.; Figure S3. Agarase sequence coverage obtained by nano LC-MS/MS Triple TOF analysis.; Figure S4. Predicted models of agarase. [file rsob190201supp1.pdf]

Supplementary Material to the article

Functional identification of a *Streptomyces lividans* FKBP-like protein involved in the folding of overproduced secreted proteins.

Vicente RL[1], Marín S[1], Valverde JR[2], Palomino C[1], Mellado RP[1], Gullón S[1]*

[1]Departamento de Biotecnología Microbiana. Centro Nacional de Biotecnología (CNB-CSIC). Darwin 3. 28049 Madrid. Spain.

[2]Scientific Computing Service, Centro Nacional de Biotecnología (CNB-CSIC). Darwin 3. 28049 Madrid. Spain.

****Corresponding author:** Sonia Gullón Blanco
Centro Nacional de Biotecnología (CSIC)
c/ Darwin 3. 28049 Madrid. Spain
Phone: +34915854523
Fax: +34915854506
E-mail: sgullon@cnb.csic.es

A

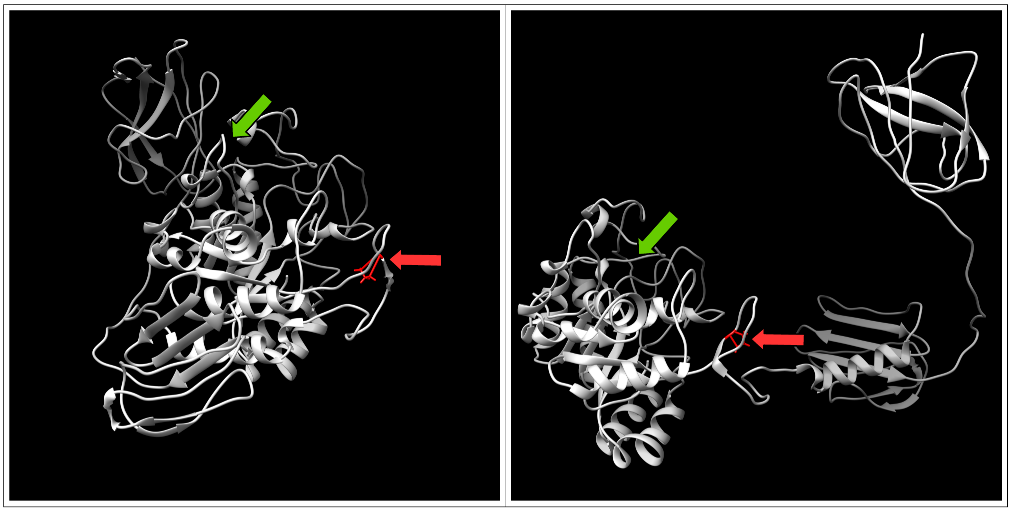

B

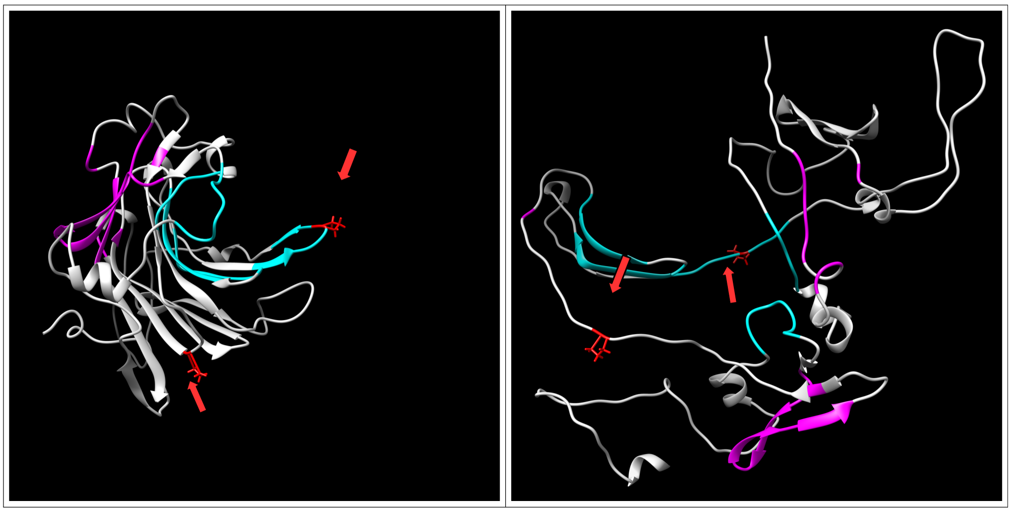

Figure S1. **Alpha-amylase and agarase models.** (A) Left: model of mature alpha-amylase with Pro350 residue in cis conformation. Right, model of immature amylase with P350 in trans conformation. The alpha-amylase Pro350 residue has been coloured in red and indicated by a red arrow. The active site is indicated by a green arrow. The cis-isomerisation of the Pro residue favours the enzyme to acquire its active conformation. (B) Left: model of mature agarase with P127 and P183 in cis conformation. Right: model of immature agarase with P127 and P183 in trans conformation. Prolines P127 and P183 are shown in red and indicated by red arrows, the active site has been coloured cyan, and the allosteric site in magenta. The models were produced using I- TASSER (alpha-amylase) or Raptor-X (agarase) and UCSF chimera, and optimized with the Amber force field.

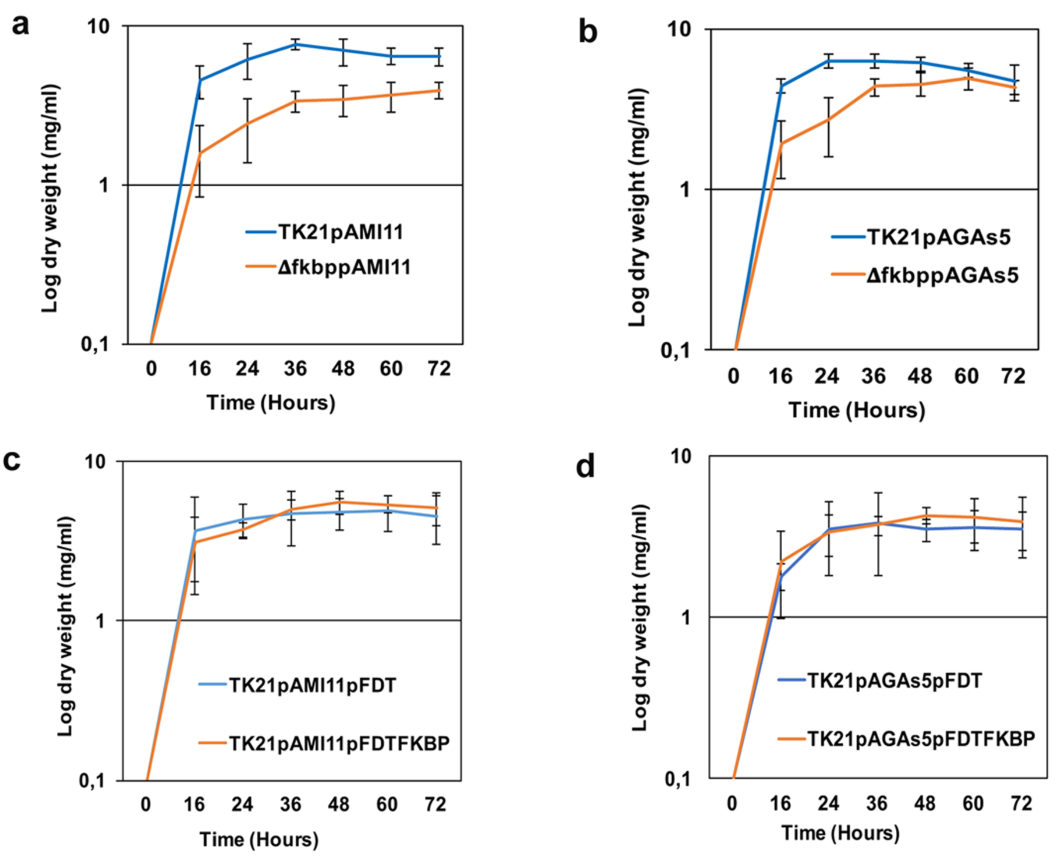

Figure S2. Growth curves of the *sli-fkbp* mutant and Sli-FKBP overproducer strains overexpressing *amlB* and *dagA*. Time course of the *S. lividans* TK21(pAMI11) and *S.lividans* Δ*sli-fkbp* (pAMI11) (a) *S. lividans* TK21(pAGAs5) and *S.lividans* Δ*sli-fkbp* (pAGAs5) (b) *S.lividans* TK21(pAMI11) (pFDT) and *S.lividans* TK21(pAMI11) (pFDTFKBP) (c) *S.lividans* TK21(pAGAs5) (pFDT) and *S.lividans* TK21(pAGAs5) (pFDTFKBP) (d).

ADLEWEQYPVPAAPGGN**RSWQLLPSHSDDFNYTGKPQTFRGRWLDQHKDG WSGPANSLYSARHSWVADGNLIVEGR**RAPDG**RVYCGYVTSRTPVEYPLYT EVLMR**VSGL**KLSSNFWLLSRDDVNEIDVIECYGNESLHGKHMNTAYHIFQR NPFTELAR**SQKGYFADGSYGYNGETGQVFGDGAGQPLLRNGFHRYGVHWISA TEFDFYFNGRLVRRLN**RSNDLRDPRSRFFDQPMHLILNTESHQWR**VD**RGIEP TDAELADPSINNIYYR**WVRTYQAV

Figure S3. Agarase sequence coverage obtained by nano LC-MS/MS Triple TOF analysis. Band corresponding to the protein with a faster mobility when Sli-FKBP was overproduced respect to the wild type was sliced out of the gel and subject to trypsin digestion and analysed by nano LC-MS/MS Triple Tof. Sequences from the mature agarase in **bold** were identified in the experiment. Yellow letters indicate catalytic residues.

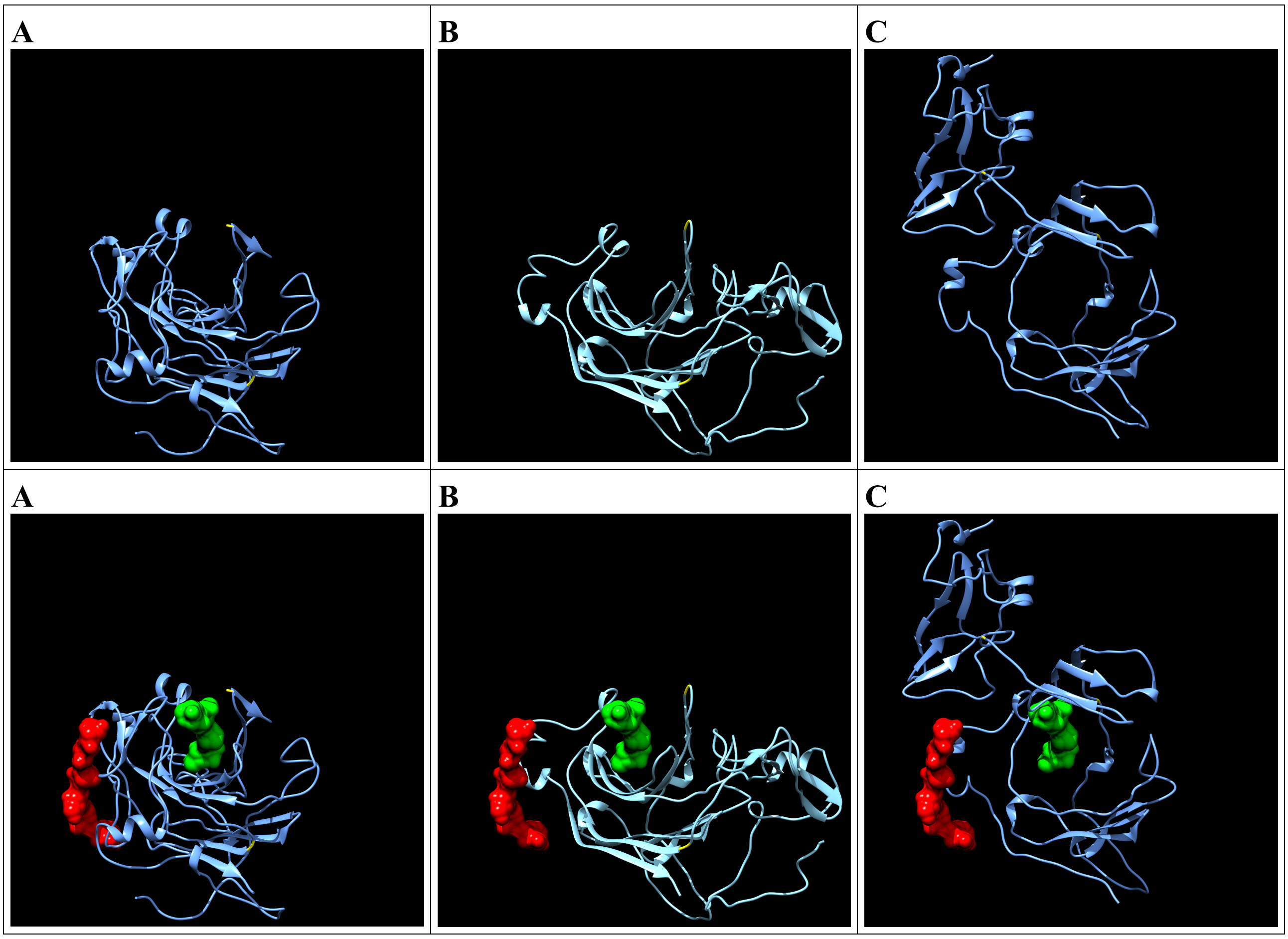

Figure S4. Predicted models of agarase.
Top row, left to right: A, mature agarase with both P127 and P183 in *cis* conformation. B, agarase with P127 in *trans* and P183 in *cis* conformation. C, agarase with both P127 and P183 in *trans* conformation. All the structures are shown using the same orientation to show the conservation of the active site in the P127*trans*, P183*cis* conformation. In the lower row we have superposed agarose bound to the active site (green) and the allosteric processive site (red) to illustrate the suggested disruption of the allosteric site in both *trans* conformations (B, C) and of the active site in the double *trans* conformer (C).
