# Supplementary material for: Functional identification of a Streptomyces lividans FKBP-like protein involved in the folding of overproduced secreted proteins
Source: Open Biol. 2019 Oct 30;9(10):190201. doi: 10.1098/rsob.190201 (PMC6833217; doi:10.1098/rsob.190201)
Supplement: Reviewer comments [file rsob190201_review_history.pdf]

THE ROYAL SOCIETY
PUBLISHING

OPEN
BIOLOGY

Functional identification of a *Streptomyces lividans* FKBP-like protein involved in the folding of overproduced secreted proteins

R. L. Vicente, S. Marín, J. R. Valverde, C. Palomino, R. P. Mellado and S. Gullón

Article citation details
Open Biol. **9**: 190201.
http://dx.doi.org/10.1098/rsob.190201

Review timeline

Original submission:	16 January 2019
1st revised submission:	21 August 2019
2nd revised submission:	1 October 2019
Final acceptance:	1 October 2019

Note: Reports are unedited and appear as submitted by the referee. The review history appears in chronological order.

Review History

RSOB-19-0013.R0 (Original submission)

Review form: Reviewer 1

Recommendation
Reject – article is not of sufficient interest (we will consider a transfer to another journal)

Are each of the following suitable for general readers?

a) **Title**
Yes

b) **Summary**
Yes

c) **Introduction**
Yes

Reports © 2019 The Reviewers; Decision Letters © 2019 The Reviewers and Editors; Responses © 2019 The Reviewers, Editors and Authors. Published by the Royal Society under the terms of the Creative Commons Attribution License http://creativecommons.org/licenses/by/4.0/, which permits unrestricted use, provided the original author and source are credited

Is the length of the paper justified?
Yes

Should the paper be seen by a specialist statistical reviewer?
No

Is it clear how to make all supporting data available?
Not Applicable

Is the supplementary material necessary; and if so is it adequate and clear?
Not Applicable

Do you have any ethical concerns with this paper?
No

Comments to the Author
See attached file

Review form: Reviewer 2

Recommendation
Major revision is needed (please make suggestions in comments)

Are each of the following suitable for general readers?

a) **Title**
Yes

b) **Summary**
Yes

c) **Introduction**
Yes

Is the length of the paper justified?
Yes

Should the paper be seen by a specialist statistical reviewer?
No

Is it clear how to make all supporting data available?
Yes

Is the supplementary material necessary; and if so is it adequate and clear?
Not Applicable

Do you have any ethical concerns with this paper?
No

Comments to the Author
Vicente et al describe the identification of a FKBP-like protein in Streptomyces lividans that plays a role in the production of increased amounts of active overproduced secreted proteins.
This is well written paper with a straightforward observation. From a biotechnological perspective the findings will be useful in protein secretion applications. What the paper is missing is sufficient biological insight that the authors could provide. Currently the paper is phenomenological without any direct molecular explanation as to what causes the changes they see and even if they are direct.

Major
1. The authors mention that western blots (not shown) showed no enhancement in levels of the secreted enzymes but only in activities. I presume this is why they attribute their findings to a direct effect of the FKBP-like protein on folding of the already secreted proteins. However, their data do not provide any direct evidence for a direct effect on the secreted reporters and are rather phenomenological. What is the evidence that the FKBP-like protein does not operate indirectly by modifying a secretion component for example or another chaperone??
The same is true for the deletion experiment. What is the evidence that FKBP-like protein is not needed for asome generic cell structure/export job that is compromised?
2. What is the effect of deleting or over-expressing the FKBP-like protein on the secretome over-all?? Can it be claimed for its role to be rather generic or is it specialized??
3. I am afraid there is no justification for the structures in Fig. 4 as the indicated Pro residues are completely speculative. In their current state these figures belong to a supplement. Otherwise, the authors need to mutate these residues and demonstrate folding effects.
4. The discussion essentially repeats the results. Its should stick to real discussion issues and be significantly trimmed.
5. How are the units normalized in the experiments? Are the activities expressed per cell?? Per fixed DCW?? Do the cells grow to the same extent when the FKBP-like protein gene is either deleted or over-expressed?

Minor grammar editing
249 were subtracted FROM those obtained in the presence of pIJ486 protoplasts and pIJFKBP

261 the secretion stress response S. lividans TK21, which consists OF the induction of the

268 genes was determined by qRT-PCR analyses and compared TO that of the wild type cell. A

278 extracellular alpha-amylase severely decreased WITH respect to that of the wild type (Fig 2A).

282 mutant when compared to that of the wild type (Fig 2B), although TO a lesser extent THAN that (or THAN IN)

288 amylase activity increased, around three-fold WITH respect TO the wild type overproducing

302 dependent agarase is overproduced, IT needs the action of the isomerase to improve its

Decision letter (RSOB-19-0013.R0)

27-Feb-2019

Dear Dr Mellado,

We are writing to inform you that your manuscript RSOB-19-0013 entitled "Functional identification of a Streptomyces lividans FKBP-like protein involved in the folding of overproduced secreted proteins." has, in its current form, been rejected for publication in Open Biology.

The referees have recommended that major revisions are necessary but that the manuscript has potential; hence, we would like to actively encourage you to revise the manuscript accordingly, and resubmit. Nevertheless, please note that this is not a provisional acceptance.

The resubmission will be treated as a new manuscript and will re-enter the review process. Every attempt will be made to use the original referees, but this cannot be guaranteed. Please note that resubmissions must be submitted within six months of the date of this email. In exceptional circumstances, extensions may be possible if agreed with the Editorial Office. Manuscripts submitted after this date will be automatically rejected.

Please find below the comments made by the referees, not including confidential reports to the Editor, which I hope you will find useful. If you do choose to resubmit your manuscript, please upload a 'response to referees' document including details of how you have responded to the comments, and the adjustments you have made.

To upload a resubmitted manuscript, log into http://mc.manuscriptcentral.com/rsob and enter your Author Centre, where you will find your manuscript title listed under "Manuscripts with Decisions." Under "Actions," click on "Create a Resubmission." Please be sure to indicate in your cover letter that it is a resubmission, and supply the previous reference number.

Sincerely,
The Open Biology Team
mailto: openbiology@royalsociety.org

Associate Editor, Professor J Peter Young
Board Member:
Comments to Author:
This manuscript has been reviewed by two experts who agree that the findings are interesting and could make a good paper, but considerable revision and the addition of new material is necessary. Each of the reviewers makes a series of good points that you need to consider and respond to. Both point out that important conclusions are drawn from Western blots that are not presented in the manuscript. They must be included. Furthermore, both reviewers are concerned that you do not present any evidence for direct interactions between Fkbp and the substrates, and they point out a number of ways in which the results could be generated by indirect effects. You must provide additional data to address this issue, but I leave it to you to decide on the best experiments that will convince the reviewers that your proposed mechanism is correct.

Reviewer(s)' Comments to Author(s):
Referee: 1
Comments to the Author(s)
See attached file

Referee: 2

Comments to the Author(s)
Vicente et al describe the identification of a FKBP-like protein in Streptomyces lividans that plays a role in the production of increased amounts of active overproduced secreted proteins.
This is well written paper with a straightforward observation. From a biotechnological perspective the findings will be useful in protein secretion applications. What the paper is missing is sufficient biological insight that the authors could provide. Currently the paper is phenomenological without any direct molecular explanation as to what causes the changes they see and even if they are direct.

Major
1. The authors mention that western blots (not shown) showed no enhancement in levels of the secreted enzymes but only in activities. I presume this is why they attribute their findings to a direct effect of the FKBP-like protein on folding of the already secreted proteins. However, their data do not provide any direct evidence for a direct effect on the secreted reporters and are rather phenomenological. What is the evidence that the FKBP-like protein does not operate indirectly by modifying a secretion component for example or another chaperone??
The same is true for the deletion experiment. What is the evidence that FKBP-like protein is not needed for asome generic cell structure/export job that is compromised?
2. What is the effect of deleting or over-expressing the FKBP-like protein on the secretome over-all?? Can it be claimed for its role to be rather generic or is it specialized??
3. I am afraid there is no justification for the structures in Fig. 4 as the indicated Pro residues are completely speculative. In their current state these figures belong to a supplement. Otherwise, the authors need to mutate these residues and demonstrate folding effects.
4. The discussion essentially repeats the results. Its should stick to real discussion issues and be significantly trimmed.
5. How are the units normalized in the experiments? Are the activities expressed per cell?? Per fixed DCW?? Do the cells grow to the same extent when the FKBP-like protein gene is either deleted or over-expressed?

Minor grammar editing
249 were subtracted FROM those obtained in the presence of pIJ486 protoplasts and pIJFKBP

261 the secretion stress response S. lividans TK21, which consists OF the induction of the

268 genes was determined by qRT-PCR analyses and compared TO that of the wild type cell. A

278 extracellular alpha-amylase severely decreased WITH respect to that of the wild type (Fig 2A).

282 mutant when compared to that of the wild type (Fig 2B), although TO a lesser extent THAN that (or THAN IN)

288 amylase activity increased, around three-fold WITH respect TO the wild type overproducing

302 dependent agarase is overproduced, IT needs the action of the isomerase to improve its

Author's Response to Decision Letter for (RSOB-19-0013.R0)

See Appendix A.

RSOB-19-0201.R0

Review form: Reviewer 1

Recommendation
Accept with minor revision (please list in comments)

Do you have any ethical concerns with this paper?
No

Comments to the Author
Major Comments
1. Line 321: Inhibition of the Fkbp protein is discussed. However, the data isn't represented anywhere. Why has this not been included in Fig1 (b) for example? Its highly relevant data.
2. Line 337: qRT-PCR analysis is discussed. However, the data isn't represented anywhere in the tables/figures. Why has this not been included in the main body or as supplementary data?
3. Line 355 and Line 369: the sentence is slightly misleading, as it implies that Fig 2 in the main text shows growth rate. This is only shown clearly in Supplementary Fig 2.
4. Line 371 and 383: The respective words 'require' and 'need' are strong here. Considering Tat-proteins are pre-folded and clearly do not require PKBP activity for normal functioning (shown in Fig 2). Consider a milder term such as overexpressed amylase 'benefits' from PKBP.
5. Section starting Line 416-458: This section needs to be re-written, in its current form its very confusing. Please explain what is meant by the Sli-PKBP control and why is FKBP detected in the mutant fkbp strain (5509 relative abundance?). Line 433: Sentence starting 'in some cases' doesn't make sense. Line 445: It is not clear to me why S. lividans TK24 and TK21 are discussed here and what the annotation means, what is meant by strain of origin? Table 1: (1) Could you show the actual ratio of relative abundance between the strains instead of only the p-value?

Minor comments
1. Line 287: Heading says Characterisation – data discussed is more identification of fkbp gene.
2. Line 345: This paragraph suddenly starts taking about amylase and agarase, whereas previous paragraph discussed secretion stress response. It feels like a heading is missing here.
3. Line 399: This is the first mention of molecular dynamics in the main text, introduce what MD stands for ease of the reader.
4. Line 468: Figure 1 doesn't show inhibition, this is misleading sentence construction. (See major comment 1)
5. Line 480, 492: The use of 'required and 'need' . These sentences are misleading in that it implies Agarase requires the action of FKBP for maturation. This is a Tat-dependent substrate and under normal conditions is secreted in its mature form without FKBP action. Consider a milder term

Decision letter (RSOB-19-0201.R0)

22-Sep-2019

Dear Dr Gullón,

We are pleased to inform you that your manuscript RSOB-19-0201 entitled "Functional identification of a Streptomyces lividans FKBP-like protein involved in the folding of

overproduced secreted proteins." has been accepted by the Editor for publication in Open Biology. The reviewer(s) have recommended publication, but also suggest some minor revisions to your manuscript. Therefore, we invite you to respond to the reviewer(s)' comments and revise your manuscript.

Please submit the revised version of your manuscript within 7 days. If you do not think you will be able to meet this date please let us know immediately and we can extend this deadline for you.

To revise your manuscript, log into https://mc.manuscriptcentral.com/rsob and enter your Author Centre, where you will find your manuscript title listed under "Manuscripts with Decisions." Under "Actions," click on "Create a Revision." Your manuscript number has been appended to denote a revision.

You will be unable to make your revisions on the originally submitted version of the manuscript. Instead, please revise your manuscript and upload a new version through your Author Centre.

When submitting your revised manuscript, you will be able to respond to the comments made by the referee(s) and upload a file "Response to Referees" in "Section 6 - File Upload". You can use this to document any changes you make to the original manuscript. In order to expedite the processing of the revised manuscript, please be as specific as possible in your response to the referee(s).

Please see our detailed instructions for revision requirements https://royalsociety.org/journals/authors/author-guidelines/.

Before uploading your revised files please make sure that you have:

1) A text file of the manuscript (doc, txt, rtf or tex), including the references, tables (including captions) and figure captions. Please remove any tracked changes from the text before submission. PDF files are not an accepted format for the "Main Document".

2) A separate electronic file of each figure (tiff, EPS or print-quality PDF preferred). The format should be produced directly from original creation package, or original software format. Please note that PowerPoint files are not accepted.

3) Electronic supplementary material: this should be contained in a separate file from the main text and meet our ESM criteria (see http://royalsocietypublishing.org/instructions-authors#question5). All supplementary materials accompanying an accepted article will be treated as in their final form. They will be published alongside the paper on the journal website and posted on the online figshare repository. Files on figshare will be made available approximately one week before the accompanying article so that the supplementary material can be attributed a unique DOI.

Online supplementary material will also carry the title and description provided during submission, so please ensure these are accurate and informative. Note that the Royal Society will not edit or typeset supplementary material and it will be hosted as provided. Please ensure that the supplementary material includes the paper details (authors, title, journal name, article DOI). Your article DOI will be 10.1098/rsob.2016[last 4 digits of e.g. 10.1098/rsob.20160049].

4) A media summary: a short non-technical summary (up to 100 words) of the key findings/importance of your manuscript. Please try to write in simple English, avoid jargon, explain the importance of the topic, outline the main implications and describe why this topic is newsworthy.

Images
We require suitable relevant images to appear alongside published articles. Do you have an image we could use? Images should have a resolution of at least 300 dpi, if possible.

Data-Sharing
It is a condition of publication that data supporting your paper are made available. Data should be made available either in the electronic supplementary material or through an appropriate repository. Details of how to access data should be included in your paper. Please see http://royalsocietypublishing.org/site/authors/policy.xhtml#question6 for more details.

Data accessibility section
To ensure archived data are available to readers, authors should include a 'data accessibility' section immediately after the acknowledgements section. This should list the database and accession number for all data from the article that has been made publicly available, for instance:
- DNA sequences: Genbank accessions F234391-F234402
- Phylogenetic data: TreeBASE accession number S9123
- Final DNA sequence assembly uploaded as online supplemental material
- Climate data and MaxEnt input files: Dryad doi:10.5521/dryad.12311

Once again, thank you for submitting your manuscript to Open Biology, we look forward to receiving your revision. If you have any questions at all, please do not hesitate to get in touch.

Sincerely,
The Open Biology Team
mailto:openbiology@royalsociety.org

Reviewer(s)' Comments to Author:
Referee: 1

Comments to the Author(s)
Major Comments
1. Line 321: Inhibition of the Fkbp protein is discussed. However, the data isn't represented anywhere. Why has this not been included in Fig1 (b) for example? Its highly relevant data.
2. Line 337: qRT-PCR analysis is discussed. However, the data isn't represented anywhere in the tables/figures. Why has this not been included in the main body or as supplementary data?
3. Line 355 and Line 369: the sentence is slightly misleading, as it implies that Fig 2 in the main text shows growth rate. This is only shown clearly in Supplementary Fig 2.
4. Line 371 and 383: The respective words 'require' and 'need' are strong here. Considering Tat-proteins are pre-folded and clearly do not require PKBP activity for normal functioning (shown in Fig 2). Consider a milder term such as overexpressed amylase 'benefits' from PKBP.
5. Section starting Line 416-458: This section needs to be re-written, in its current form its very confusing. Please explain what is meant by the Sli-PKBP control and why is FKBP detected in the mutant fkbp strain (5509 relative abundance?). Line 433: Sentence starting 'in some cases' doesn't make sense. Line 445: It is not clear to me why S. lividans TK24 and TK21 are discussed here and what the annotation means, what is meant by strain of origin? Table 1: (1) Could you show the actual ratio of relative abundance between the strains instead of only the p-value?

Minor comments
1. Line 287: Heading says Characterisation – data discussed is more identification of fkbp gene.
2. Line 345: This paragraph suddenly starts taking about amylase and agarase, whereas previous paragraph discussed secretion stress response. It feels like a heading is missing here.

3. Line 399: This is the first mention of molecular dynamics in the main text, introduce what MD stands for ease of the reader.
4. Line 468: Figure 1 doesn't show inhibition, this is misleading sentence construction. (See major comment 1)
5. Line 480, 492: The use of 'required and 'need' . These sentences are misleading in that it implies Agarase requires the action of FKBP for maturation. This is a Tat-dependent substrate and under normal conditions is secreted in its mature form without FKBP action. Consider a milder term

Author's Response to Decision Letter for (RSOB-19-0201.R0)

See Appendix B.

Decision letter (RSOB-19-0201.R1)

01-Oct-2019

Dear Dr Gullón

We are pleased to inform you that your manuscript entitled "Functional identification of a Streptomyces lividans FKBP-like protein involved in the folding of overproduced secreted proteins." has been accepted by the Editor for publication in Open Biology.

You can expect to receive a proof of your article from our Production office in due course, please check your spam filter if you do not receive it within the next 10 working days. Please let us know if you are likely to be away from e-mail contact during this time.

Article processing charge
Please note that the article processing charge is immediately payable. A separate email will be sent out shortly to confirm the charge due. The preferred payment method is by credit card; however, other payment options are available.

Thank you for your fine contribution. On behalf of the Editors of Open Biology, we look forward to your continued contributions to the journal.

Sincerely,

The Open Biology Team
mailto: openbiology@royalsociety.org

Appendix A

Referee 2

General remarks.

Point 1. What is the evidence that the FKBP-like protein does not operate indirectly by modifying a secretion component for or another chaperone? What is the evidence that FKBP-like protein is not needed for asome generic cell structure/export job that is compromised?

Response: Recently, it has been reported the *S. lividans* TK24 secretome showing that SLIV_29545 is the only extracytoplasmic PPIase. We performed targeted mass spectrometry analyses (SRM/MRM) to study the relative protein abundance of a SLIV_18490 that has been annotated in the secretome of *S. lividans* TK24 as chaperone protein and the extracytoplasmic secretion components involved in the folding previously identified in our group ([24, 58]; using supernatants of the Δ*sli-fkbp*, the single-copy *sli-fkbp* and the *sli-fkbp* overexpressing strains. Results are summarised in a new Table1 and Table S1 and in the Results section of the new version of the manuscript page 21 lines 451-458 and in the Discussion section of the new version of the manuscript page 24-25 lines 539 to 555.

Briefly, secretion is not compromised in a mutant lacking FKBP, although the amount of correctly folded proteins seems to be.

Point 2. What is the effect of deleting or over-expressing the FKBP-like protein on the secretome over-all? Can it be claimed for its role to be rather generic or is it specialized?

Response: We have performed targeted mass spectrometry (SRM/MRM) analyses to study the effect of the deletion or overexpression of SLI-FKBP over a group of selected proteins potentially isomerised by Sli-FKBP showing that Sli-FKBP affects a relevant group of secreted proteins. The results are summarised in a new Table 1, Table S1 and are mentioned in the Result section page 18 lines 416-442 and in the Discussion section page 24 lines 531-534 showing that Sli-FKBP may have a wide substrate range.

Point 3. I am afraid there is no justification for the structures in Fig 4. As the indicated Pro residues are completely speculative. In their current state these figures belong to a supplement. Otherwise, the authors need to mutate these residues and demonstrate folding effects.

Response: In the new version of the manuscript the former Fig 4 has been moved to the supplementary material (figure S1).

Point 4. The discussion essentially repeats the results. Its should stick to real discussion issues and be significantly trimmed.

Response: Thank you for the suggestion. The discussion has been rewritten according to your recommendation.

Point 5. How are the units normalized in the experiments? Are the activities expressed per cell?? Per fixed DCW?? Do the cell grow to the same extent when the FKBP-like protein gene is either deleted or over-expressed?

Response: When the FKBP-like gene is deleted the growth has been affected, mainly in the Δ*sli-fkbp* mutant strain overproducing alpha-amylase. Nevertheless, overproduction of Sli-FKBP did not affect significantly the rate of growth in liquid medium. These

results have been included in a new Fig S2 and in the Results section of the new version of the manuscript page 15 lines 355-355 and page 16 lines 368-369.
According to this, the equivalent amount of protein loaded onto the SDS-PAGE acrylamide gel was corrected by the bacterial dry weight in each case. A new sentence has been added in the Methods section page 8 lines 167-169. Alpha-amylase activity was normalized per mg of protein and agarase activity was normalized per mg of dry weight.

Point 7. Minor grammar editing

Line 249 Thank you for the correction of has been replaced by from
Line 261 This sentence has been removed according to reviewer 1 suggestion.
Line 268 “to” has been added.
Line 278 “with” has been added.
Line 282 “in” has been removed and by “to a lesser extent than” has been added.
Line 288 “with respect to” has been added.
Line 302 “it” has been included.

Referee 1

1. Protein prediction study describe in Materials and Methods, presented in Figure 4 and discussed in Line 333-337 is not mention of in any of the results sections.
   Response: The former Figure 4 in the new version of the manuscript has been moved to the supplementary material (figure S1) according to the suggestion of reviewer 2. This figure is mentioned in the Results section of the new version of the manuscript page 15 lines 345-348 and also in the Discussion section page 22 lines 479-483.

2. Novelty-PrsA does similar things. Its not explained why this protein is particular is novel.
   Response: Analysis of the *S. lividans* secretome [59 of the new version of the manuscript] shows that the only extracytoplasmic PPIase in *S. lividans* is this one. Sli-FKBP is an homolog to FKBP and thus should be classified in the family of FK506 binding proteins, while PrsA is a parvulin, hence belonging to a different family in a different organism. The findings in *B. subtilis* therefore, cannot be applied to *S. lividans* where no parvulin-like proteins have been identified in the secretome. This is the first report of such an extracytoplasmic PPIase involved in the folding of secreted proteins in *Streptomyces* and the results obtained provide useful information for use in engineering *Streptomyces* strains for homologous or heterologous secretory protein overproduction.

3. Line 253: An experiment describing the inhibition of FKBP using FK506 is described but the data for this is not shown. This is an important conclusion and needs to be included in the results section. It is also mentioned in the discussion Line 311, however, since this information is not actually presented it shouldn’t be used to make such conclusions.
   Response: Data from inhibition assays has now been included in the results section of the new version of the manuscript page 14 lines 321-328.

4. Line 276-283. The activity of amylase and agarase are measured and shown to decrease when Fkbp is absent, activity assays are excellent methods of showing that the deleted Fkbp is linked to the activity of Amylase and Agarase. However, it is implied that the reason the Agarase/amylase activity is decreased is due to an increase in misfolded protein in the absence of the Fkbp. However, its not clear that in the absence of Fkbp the same amount of Sec/Tat substrate is actually secreted. Western blot assays for Amylase and Agarase could show whether the amount of protein present is altered.
   Response: Thank you for the suggestion. A new figure 2 including western blot of the Δ*sli-fkbp* mutant overproducing alpha-amylase and agarase has been included showing that the alpha-amylase secretion pattern has been severely affected.

5. Line 294 and Line 304. The authors say that Western blot assays were performed and showed that increased Amylase/Agarase production remained the same with or without Fkbp. The reference another paper and do not shown the data. Western blot showing this information is essential to making this statement, further this is an exceptionally important observation that needs to be incorporated into the results.
   Response: A new figure 3 that compares the Sli-FKBP and agarase or alpha-amylase overproducing strains with their isogenic strains, TK21pAMI11pFDT (alpha-amylase) and TK21pAGAs5pFDT (agarase) has been added to the manuscript. It reveals that overproduction of Sli-FKBP improves alpha-amylase secretion although there is no significant increase in extracellular production of agarase despite the increase of activity.

6. The absence of Fkbp has an effect on Sec and Tat secreted proteins. However, direct interactions between Fkbp and these substrates have not been shown. This is particularly important with regard to the Tat substrate, as these are secreted in a folded form. The effect that Fkbp has on Agarase activity might not be directly associated with Fkbp but rather an indirect effect of Fkbp on membrane protein stability/decrease stress response (a more stable environment results in more active protein). This is not addressed in the results section or the discussion. In Line 301 it's also said that Fkbp isomerase action improves Tat-dependent agarase folding. Direct protein-protein interaction studies such as co-precipitation studies would show this interaction.
   Response: We do agree that more experiments showing protein-protein interactions are needed but at present this is beyond our possibilities. The new version of the manuscript presents the study based on agarase modelling (new figure S4) as a potential explanation without asserting it. New text has been included in Results section page 17-18 lines 396-415 and Discussion section page 24 lines 521 and 527.

Minor comments:

1. Line 59. It is not immediately clear to the reader that the Sec pathway is being described when referring to SRP. By moving the sentence of starting in line 62 upwards this would remove that ambiguity and assist readers who are not well versed in protein secretion.
   Response: We agree with the reviewer's suggestion. The sentence has been rewritten to avoid ambiguity. New text has been included in the background section page 3 lines 58-61.

2. Line 220. In this section of the paper authors compare various FKBP-like proteins and identify/describe the gene in *S. lividans* TK21. It is not clear to me why *S. lividans* TK21 is used instead of the sequenced and standard host S. lividans TK24.
   Response: We use *S. lividans* TK21 as the reference strain in the laboratory. The choice of *S. lividans* TK21 as our reference strain is explained because we started working on protein secretion using *S. lividans* TK21 as a model organism long before the genome of *S. lividans* TK24 was available. The differences among them are minor and conclusions should be directly transferable among them.

3. Line 222 is written in such a way that it infers the function of FKBP-like protein (SCO1639) was deduced. This is not the case the references used here refer to deducing the signal peptide sequence of SCO1639 is Tat-specific, in these papers the function of the protein is only predicted. However, functional studies have been done by others eg. on orthologous *Streptomyces chrysomallus* (Pahl and Keller, 1994 EMBO J)
   Response: Thank you for pointing out this. We have rewritten the sentence according to the suggestion to avoid misunderstanding and we have introduced the reference. (Page 13, lines 289-291).

4. Line 228-230: A 1222 bp fragment was PCRed and sequenced, the authors describe this as the gene and a regulatory region. What is meant by regulatory region and how long is it?
   Response: The regulatory region is putative, we selected a region upstream of the translation start site. S1 assays must be performed to determine the specific regulatory region. To avoid misunderstandings, we introduce in the new version of the manuscript in page 13 line 298 the sentence "the potential regulatory region" of the gene.

5. The Tat-dependency of this FKBP-like protein, as shown by others, is discussed multiple times in this results section (Line 222 and is re-iterated in line 234-238). Considering this study the Tat-dependency of SLIV_29545 is not investigated these comments could be mentioned, but rather discussed further in the introduction/ discussion section.
   Response: Thank you for the suggestion. We have eliminated comments related to this in the new version of the manuscript.

6. Line 257-260. The authors describe qRT-PCR results that are not shown in the paper. In line 260-263 the authors continue to discuss secretion stress response

described in other studies. Its not clear to me why these studies are described in such detail in this section of this paper.
Response: Thank you for the suggestion. The qRTPCR transcriptional analyses of *tatC* in *S. lividans* TK21 have been previously published [26 of the new manuscript] we add a new sentence showing that the expression level of *sli-fkbp* is only 10% with respect to that of *tatC* expression. We have deleted line 260-263 of the former version of the manuscript, this information appears in the new version of the manuscript in the discussion section page 22 lines 469-472.

7. Line 266. “To further” is not accurate as this hypothesis has not been previously tested here and is superfluous.
   Response: We have modified the text according to your suggestion.

8. Line 266: please mention the king of mutant generated. From reading the materials section it looks like an antibiotic cassette was inserted into the *fkbp* gene however this is not clear.
   Response: The disrupted mutant was generated by the insertion of the plasmid pOJ260 into the *sli-fkbp* gene. New sentences have been added to clarify the construction of the mutant in the new version of the manuscript (Methods section page 5 lines 103-106 and Results section, page 15 line 336)

9. Line 275. There is a sudden jump in results presented here. The authors go on in Line 275-283 to describe an assay where two secreted proteins are expressed in high amounts in a genetic background with a mutated *sli-fkbp*. Both of Sec-and Tat-secreted products investigated showed a decrease in activity when Fkbp was absent. If the authors introduce this section better it would read easier.
   Response: This is true. A new sentence has been included in the Results section to facilitate reading (page 15 lines 349-353).

10. Line 281: “deficient strain” implies a strain where expression is diminished but not deleted/knocked-out, in this case it was a mutant strain (unless I’ve misunderstood the way the mutant was made, see Minor comment 4).
    Response: The disrupted mutant as described above was generated using a DNA internal fragment of the gene candidate to mutate, this DNA is inserted in to a vector which then integrates into the chromosome by a single homologous crossover. This approach results in an integrated copy of the vector flanked by one truncated part of the gene at the 3’ end and the other at the 5’that are not functional. This strategy has been commonly used to obtain mutants in *Streptomyces* [24, 25 of the new version of the manuscript, Thomas et al 2003, Olano C et al 2004). To avoid misinterpretation, we replace deficient strain to mutant strain throughout the manuscript.

Thomas MG, Chan YA, Ozanick SG. Deciphering tuberactinomycin biosynthesis: isolation, sequencing, and annotation of the viomycin biosynthetic gene cluster. 2003. Antimicrob Agents Chemother. 47: 2823-2830. doi: 10.1128/AAC.47.9.2823-2830.2003.

Olano C, Wilkinson B, Sánchez C, Moss SJ, Sheridan R, Math V, Weston AK, Braña AF, Martin CK, Olynyk M, Méndez C, Leadlay PF and Salas JA. Biosynthesis of the angiogenesis inhibitor borrelidin by *Streptomyces parvulus*

Tü4055: cluster analysis and assignment of functions. 2004. Chem Biol 11:87-97.

11. Line 326-332- The use of the word "despite" does not make sense to me here. As its not clear why late exponential expression would be effected by the presence of a Tat-signal peptide.
Response: Previously, we determined the expression of one of the components of the Tat route, *tatC*, revealing that it reaches its maximum expression level at the stationary phase of growth [26 of the new manuscript]. For this reason, a protein containing a Tat-signal peptide will be majorly secreted after the late exponential phase of growth. Our previous, decades long, experience in Tat secretion in *S. lividans* TK21 confirms that this is actually the case. A new sentence has been added in the new version of the manuscript in the Discussion section page 23 lines 495-497.

12. Line 329-330. Its implied that overproduction of Fkbp would result in overflow secretion via the Sec system. However, the initial requirement of the Tat system to secreted folded protein is overlooked and not discussed.
Response: According to the reviewer's suggestion a new sentence has been included in the Discussion section of the new version of the manuscript page 23 lines 499-502.

.

Appendix B

Major comments:

1. Line 321: Inhibition of the FKbp protein is discussed. However, the data isn't represented anywhere Why has this not been included in Fig 1 (b) for example? It's highly relevant data?
   Response: Thank you for the suggestion. The data has been included in a new Fig 1 b and according to this the legend of the Fig 1 has been modified in the revised version of the manuscript.

2. Line 337: qRT-PCR analysis is discussed. However, the data isn't represented anywhere in the tables/figures. Why has this not been included in the main body or as supplementary data?
   Response: Data from qRT-PCR analysis has been included in line 336 showing the relative expression levels and their standard deviations. Experiments were performed with three biological replicates. We add a sentence to clarify that the values refer to the relative expression levels of *cssS* and *cssR*)

3. Line 355 and Line 369: the sentence is slightly misleading, as it implies that Fig 2 in the main text shows growth rate. This is only shown clearly in Supplementary Fig 2.
   Response: Thank you for the comment. We agree that the sentence may be misinterpreted and we have modified the figure references to clearly indicate that they refer to figure S2 in both cases in the revised version of the manuscript.

4. Line 371 and 383. The respective words "require" and "need" are strong here. Considering that Tat-proteins are pre-folded and clearly do not require FKBP activity for normal functioning (shown in Fig 2) Consider a milder term such as overexpressed amylase "benefits" from FKBP.
   Response: Thanks for the suggestion. We have modified the text accordingly and now, "require" has been replaced by "benefit" and "may need" has been replaced by "may be aided by" in the revised version of the manuscript.

5. Section starting Line 416-458: This section needs to be re-written, in its current form its very confusing. Please explain what is meant by the Sli-FKBP control and why is FKBP detected in the mutant fkbp strain (5509 relative abundance?). Line 433: Sentence starting "in some cases" doesn't make sense. Line 445: It is not clear to me why S. lividans TK24 and TK21 are discussed here and what the annotation means, what is meant by strain of origin? Table 1: (1): Could you show the actual ratio of relative abundance between the strains instead of only the p-value?
   Response: Thank you. On re-reading the text we agree that it may be confusing. This section has been completely rewritten.
   The term "control" applied to Sli-FKBP referred to its use as a check that proteomics did detect the expected expression changes, but was likely misleading in this context and has been removed.
   The Δ*sli-fkbp* strain is a disrupted mutant, not a deletion one. In our case, only one peptide (of all that identify Sli-FKBP) is detected in very low quantity in only two replicates, which may have been translated and which may have been not totally degraded by proteases (as corresponds to a defective protein).

Line 433, “in some cases...”, refers to the fact that relative abundance cannot be calculated when the observed abundance in the Δ*sli-fkbp* strain was zero (as division by zero is undefined). We realize the wording was unfortunate and have rewritten the section to avoid misunderstandings.

Line 445, experiments were performed in *S. lividans* TK21, which shares most genes with *S. lividans* TK24 (both being close derivatives of *S. lividans* 66). Since the genome of *S. lividans* TK21 is not known, there are no specific official gene identifiers and we routinely use the *S. lividans*TK24 gene names and annotation to identify homologue genes. We agree that it may be confusing for unfamiliar readers and have rewritten the section and removed the references.

Table 1: While statistical significance is the ultimate test, we recognize that is it custom practice in the literature to report ratios, and that they may improve readability by readers. We have added the ratios to the table. Additionally, during the review process we realized that the statistical tests we applied initially were too stringent and that we could increase power and reliability. We have updated the values and the text to reflect these. Some observations having too large variability were non-significant: to avoid including potentially misleading results, ratios that were not statistically significant have been omitted.

Minor comments:

1. Line 287: Heading say Characterisation-data discussed is more identification of fkbp gene.
   Response: Thank you for the suggestion. The heading has been replaced to indicate the Identification of the fkbp gene.

2. Line 345: This paragraph suddenly starts talking about amylase and agarase, whereas previous paragraph discussed secretion stress response. It feels like a heading is missing here.
   Response: Thanks. A heading has been included according to the reviewer’s suggestion.

3. Line 399: This is the first mention of molecular dynamics in the main text, introduce what MD stands for ease of the reader
   Response: MD had been defined in M&M. We have re-defined MD here as well.

4. Line 468: Figure doesn’t show inhibition, this is misleading sentence construction. (see major comment 1)
   Response: We introduce a new figure 1b which shows the inhibition by FK506 and we have referred to this figure here.

5. Line 480, 492. The use of “required” and “need”. These sentences are misleading in that it implies Agarase requires the action of FKBP for maturation. This is a Tat-dependent substrate and under normal conditions is secreted in its mature form without FKBP action. Consider a milder term.
   Response: We have replaced both words in the revised version of the manuscript for less strong terms.
